# Metabolic profiling of *Candida* clinical isolates of different species and infection sources

**DOI:** 10.1038/s41598-020-73889-1

**Published:** 2020-10-07

**Authors:** Josidel Conceição Oliver, Luca Laghi, Carola Parolin, Claudio Foschi, Antonella Marangoni, Andrea Liberatore, Amanda Latercia Tranches Dias, Monica Cricca, Beatrice Vitali

**Affiliations:** 1 Department of Pharmacy and Biotechnology, Alma Mater Studiorum, University of Bologna, Bologna, Italy; 2 Centre of Foodomics, Department of Agro-Food Science and Technology, Alma Mater Studiorum, University of Bologna, Cesena, Italy; 3 Microbiology, DIMES, Alma Mater Studiorum, University of Bologna, Bologna, Italy; 4 Department of Microbiology and Immunology, Federal University of Alfenas, Minas Gerais, Brazil

**Keywords:** Biological techniques, Biotechnology, Microbiology, Pathogenesis

## Abstract

*Candida* species are the most common cause of opportunistic fungal infections. Rapid identification and novel approaches for the characterization of these fungi are of great interest to improve the diagnosis and the knowledge about their pathogenic properties. This study aimed to characterize clinical isolates of *Candida* spp. by proteomics (MALDI-TOF MS) and metabolomics (^1^H-NMR), and to correlate their metabolic profiles with *Candida* species, source of infection and different virulence associated parameters. In particular, 49 *Candida* strains from different sources (blood, n = 15; vagina, n = 18; respiratory tract, n = 16), belonging mainly to *C. albicans* complex (61%), *C. glabrata* (20%) and *C. parapsilosis* (12%) species were used. Several extracellular and intracellular metabolites showed significantly different concentrations among isolates recovered from different sources of infection, as well as among different *Candida* species. These metabolites were mainly related to the glycolysis or gluconeogenesis, tricarboxylic acid cycle, nucleic acid synthesis and amino acid and lipid metabolism. Moreover, we found specific metabolic fingerprints associated with the ability to form biofilm, the antifungal resistance (i.e. caspofungin and fluconazole) and the production of secreted aspartyl proteinase. In conclusion, ^1^H-NMR-based metabolomics can be useful to deepen *Candida* spp. virulence and pathogenicity properties.

## Introduction

*Candida* species are the most common cause of opportunistic fungal infections mainly in immunocompromised individuals^1^. *Candida* spp. are able to cause mucocutaneous lesions, such as oropharyngeal or vaginal candidiasis, and systemically invasive infections that are associated with high mortality^2–4^. *Candida albicans* is still the most commonly isolated species, but other non-*albicans Candida* species have been isolated from patients and drawn attention because of their resistance to antifungals^5–7^.

Different *Candida* species may have very different antifungal susceptibility profiles. In this context, it is important to obtain accurate taxonomic identification to apply appropriate treatments^8,9^. Traditionally, fungi are identified by phenotypic traits including morphology, colony appearance and pigmentation in chromogenic culture medium^8,10,11^. Recently, alternative methods for rapid identification of microorganisms have been studied, such as proteomics, using matrix-assisted laser desorption/ionization time-of-flight mass spectrometry (MALDI-TOF MS), and metabolomics by nuclear magnetic resonance (NMR)^8,12–15^. MALDI-TOF MS has short analysis time, low error rate and high precision for microorganism identification because it can discriminate between closely related and critical species, making it suitable for implementation in the field of clinical routine^12,16,17^. NMR spectroscopy generates complex data based on metabolic profiles, which can be used not only for metabolites’ mere quantification, but also for accurate identification of yeast to subspecies level^18,19^. In addition, metabolic profiles have the potential to be used as antifungal resistance markers^20^. These methods are widely used in the search for factors associated with virulence of microorganisms, besides, they can help in the differential diagnosis of clinical isolates of *Candida* spp. for appropriate treatments. In this perspective, the present study aims to characterize clinical isolates of *Candida* spp. by proteomic and metabolomic methodologies, and to correlate their metabolic profiles with *Candida* species, infection source and important virulence factors, such as the ability to form biofilm, antifungal resistance and production of secreted aspartyl proteinase (SAP).

## Results

### *Candida* species distribution and hierarchic dendrograms

Forty-nine *Candida* clinical isolates, recovered from different sources of infection (30.6% blood, 36.7% vaginal tract and 32.7% respiratory tract) during routine diagnostic procedures, were identified by proteomic characterization using MALDI-TOF MS. The distribution of *Candida* species (59.2% *C. albicans*, 20.4% *C. glabrata*, 12.2% *C. parapsilosis*, 6.2% C. *tropicalis,* 2% *C. dubliniensis*; Supplementary Table S1) reflects the one found in clinical practice, as reported by Cataldi et al.^21^.

**Figure 1:**
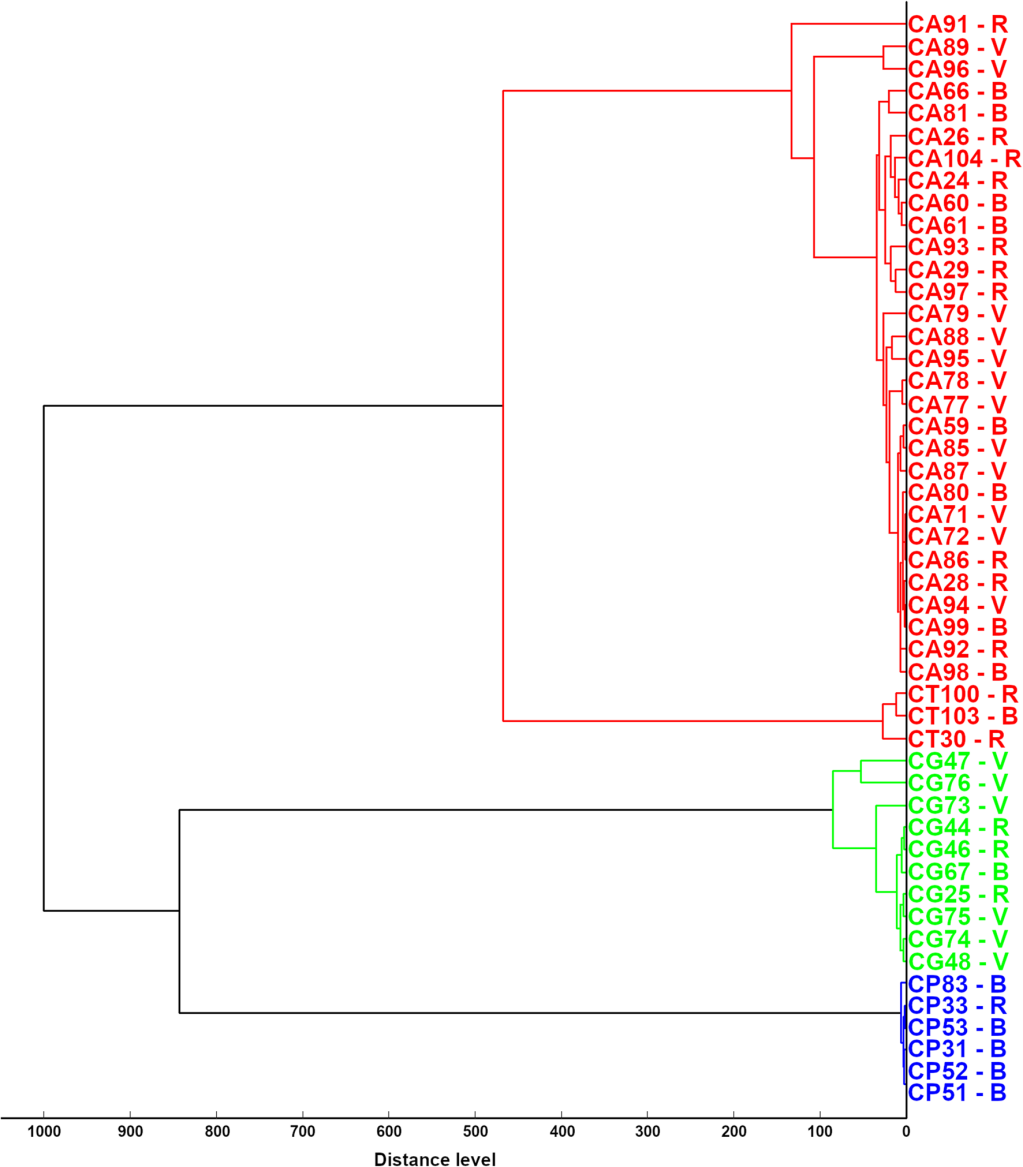
Cluster analysis of main spectrum profiles obtained by MALDI-TOF MS from *Candida* strains included in the study. In the dendrogram, relative distance between isolates is displayed as arbitrary units. Zero indicates complete similarity and 1000 indicates maximum dissimilarity. CA, *C. albicans* complex (one *C. dubliniensis* -CA91- and 29 *C. albicans*); CT, *C. tropicalis*; CG, *C. glabrata*; CP, *C. parapsilosis*, numbers identified strains. *V* isolated from vaginal tract, *B* blood origin, *R* isolated from respiratory tract.

**Figure 2:**
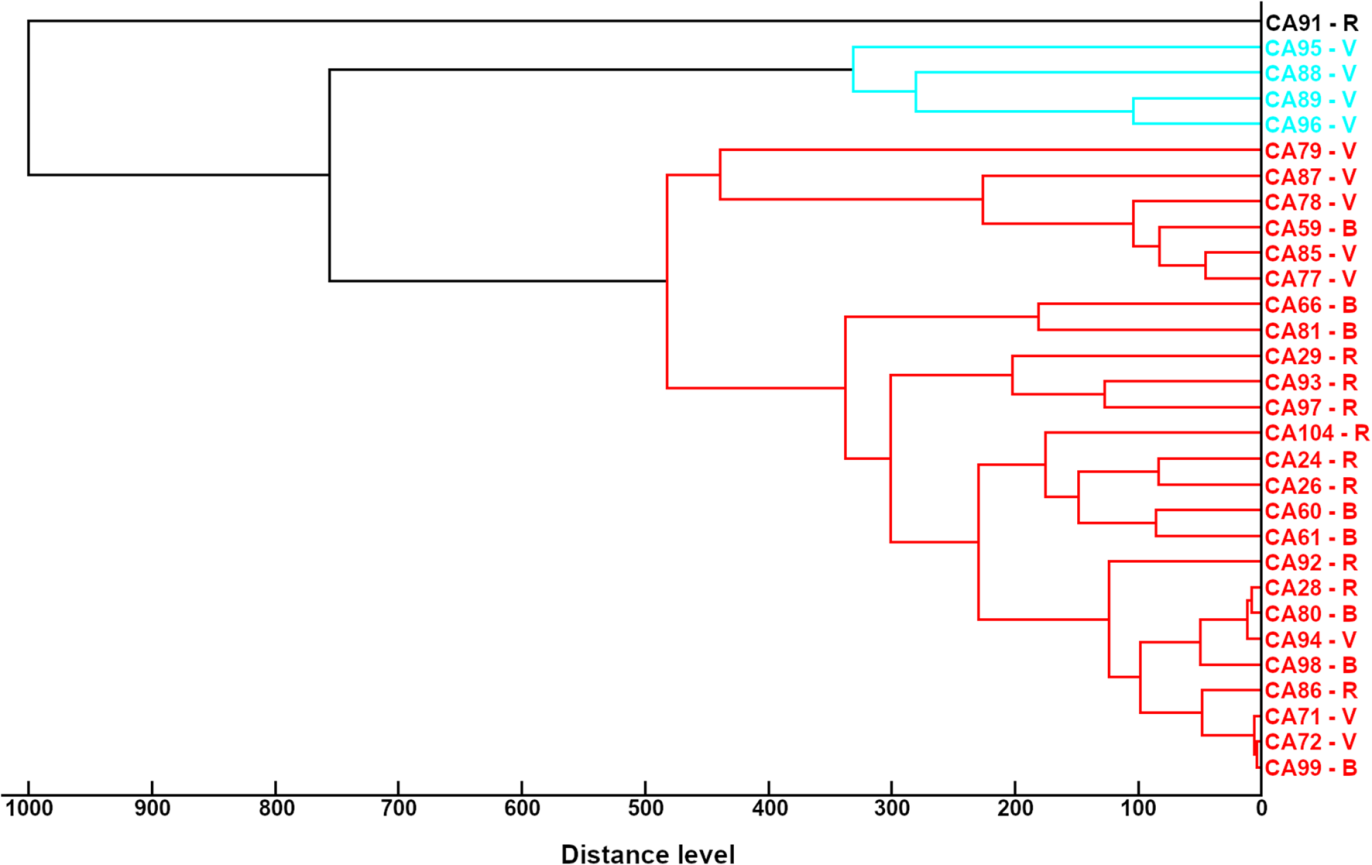
Cluster analysis of main spectrum profiles obtained by MALDI-TOF MS from *Candida albicans* strains included in the study. In the dendrogram, relative distance between isolates is displayed as arbitrary units. Zero indicates complete similarity and 1000 indicates maximum dissimilarity. CA, *C. albicans* complex, numbers identified strains. *V* isolated from vaginal tract, *B* blood origin, *R* isolated from respiratory tract.

Figure 1 shows the hierarchic dendrogram of the MSPs obtained from the 49 *Candida* strains. As expected, MSP dendrogram clustered the *Candida* strains according to their species and identified two main groups: the first one comprised *C. albicans* complex (29 *C. albicans* and one C. *dubliniensis*) and *C. tropicalis*; the second one *C. glabrata* and *C. parapsilosis*. Figure 2 shows the hierarchical dendrogram of the *C. albicans* complex, comprising 30 strains. At the distance of 1000 arbitrary units (maximum dissimilarity) *C. dubliniensis* 91 appeared as separated from all the other *C. albicans* strains, while at the distance of 760 arbitrary units it is possible to recognize two sub-groups comprising 4 and 25 *C*. *albicans* strains, respectively.

*Candida* species identification obtained by MALDI-TOF MS was always confirmed by Internal Transcribed Spacer (ITS) sequencing (Supplementary Table S1).

### Differential metabolome profiles associated with the isolation source

*Candida* strains were subjected to metabolomic analysis by ^1^H-NMR. Both *Candida* culture supernatants and cell lysates were examined, in order to depict extracellular and intracellular metabolome profiles, respectively. Metabolome analysis allowed the identification and quantification of 48 molecules in the extracellular metabolome and 47 molecules in the intracellular metabolome, with 29 molecules in common.

We evaluated whether *Candida* clinical isolates recovered from different sources of infection showed differences in their metabolome profiles. Twelve extracellular metabolites and 9 intracellular metabolites showed different concentrations when compared among isolates form blood, respiratory tract, and vagina (Supplementary Tables S2–S3). None of them could be quantified both in the extracellular and intracellular environments.

**Figure 3:**
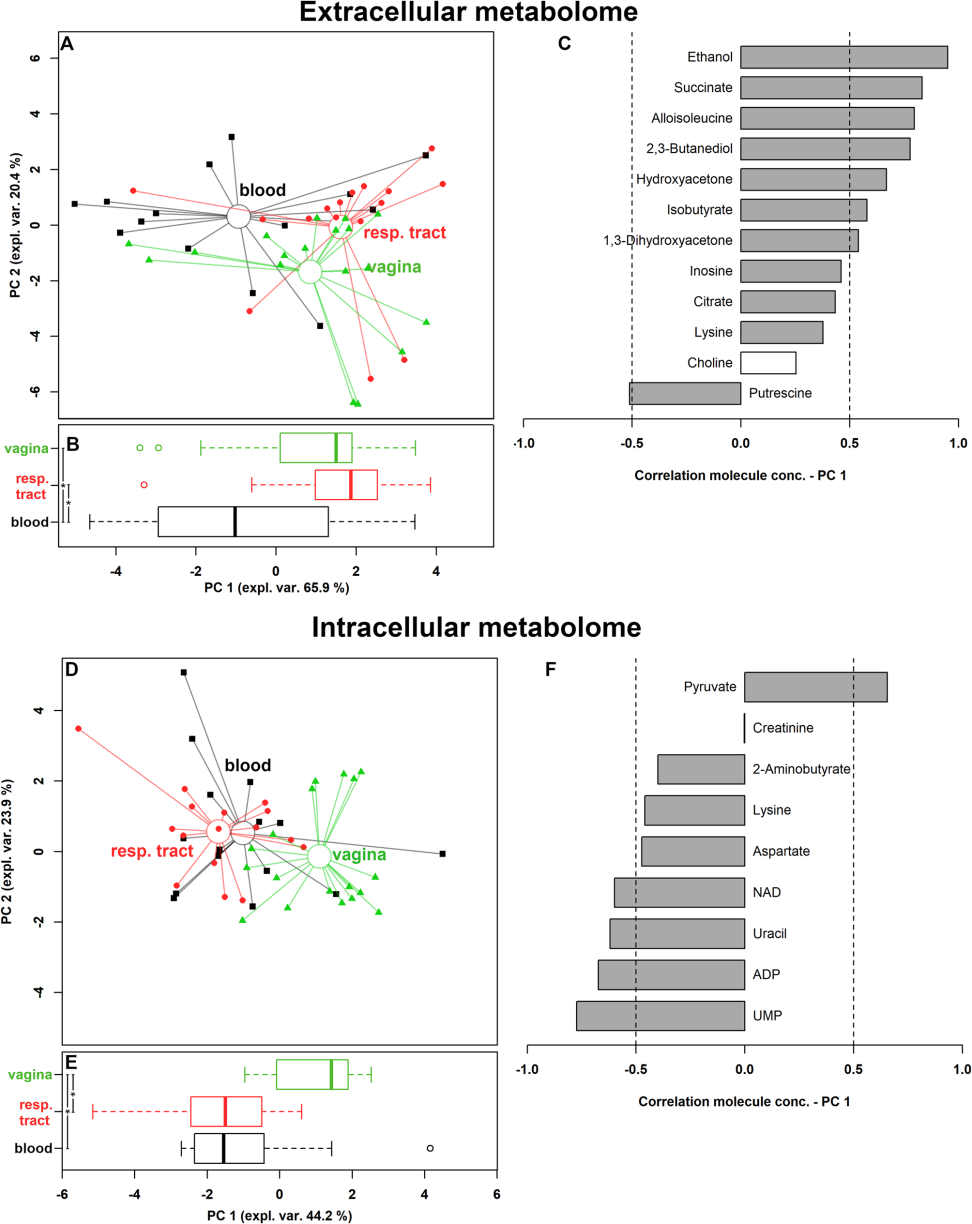
Correlation between extracellular and intracellular metabolome and *Candida* source of infection. (**A**,**D**) Scoreplots of the rPCA models calculated on the spaces constituted by the concentration of the molecules significantly different among *Candida* strains grouped on the basis of their source of infection (respiratory tract, vagina, blood). Empty circles highlight the median values of the samples from the different sources. (**B**,**E**) Box plots summarizing the positions of the samples along PC 1. (**C**,**F**) Bar plots describing the correlation between the concentration of each molecule and its importance along PC 1. *P < 0.05.

Two robust principal component analysis (rPCA) models were built to identify the overall trends underlying these molecules (Fig. 3). The data were optimally summarized by 3 and 4 principal component (PC), respectively. For both extracellular and intracellular metabolome, the first PC (PC 1) of the scoreplot offered a clear summary of the peculiarities of the strains from the different infection sources (Fig. 3A,D). Along this direction, samples collected from vagina and blood were characterized by statistically different PC 1 scores (Fig. 3B,E). The samples collected from the respiratory tract appeared similar to those collected from the vagina when the extracellular metabolome was observed, while they appeared similar to those collected from the blood when the intracellular metabolome was observed.

Figure 3C allows to visually appreciate that the molecules of the extracellular metabolome mostly contributing to the distribution of the samples along PC 1 are ethanol, succinate, alloisoleucine, 2,3-butanediol and hydroxyacetone. These molecules are produced by all the strains, particularly by those collected from the vaginal environment and the respiratory tract.

Figure 3F shows that the molecules detected in the intracellular metabolome that mostly contributed to the differentiation of the strains according to the collection site were UMP, ADP, uracil, NAD and pyruvate. UMP, ADP, uracil, NAD were more concentrated in *Candida* strains isolated from blood and respiratory tract, while pyruvate mainly characterized vaginal strains. All these molecules, together with aspartate, are involved in the metabolism of carbohydrates, whose over-representation appeared to be statistically significant (P = 0.035), as calculated by over representation analysis (ORA). A direct link between the molecules highlighted in extracellular and intracellular metabolome can be found by observing that 2,3-butanediol is produced by microorganisms from the anaerobic fermentation of pyruvate^22^.

### Differential metabolome profiles associated with *Candida* species

**Figure 4:**
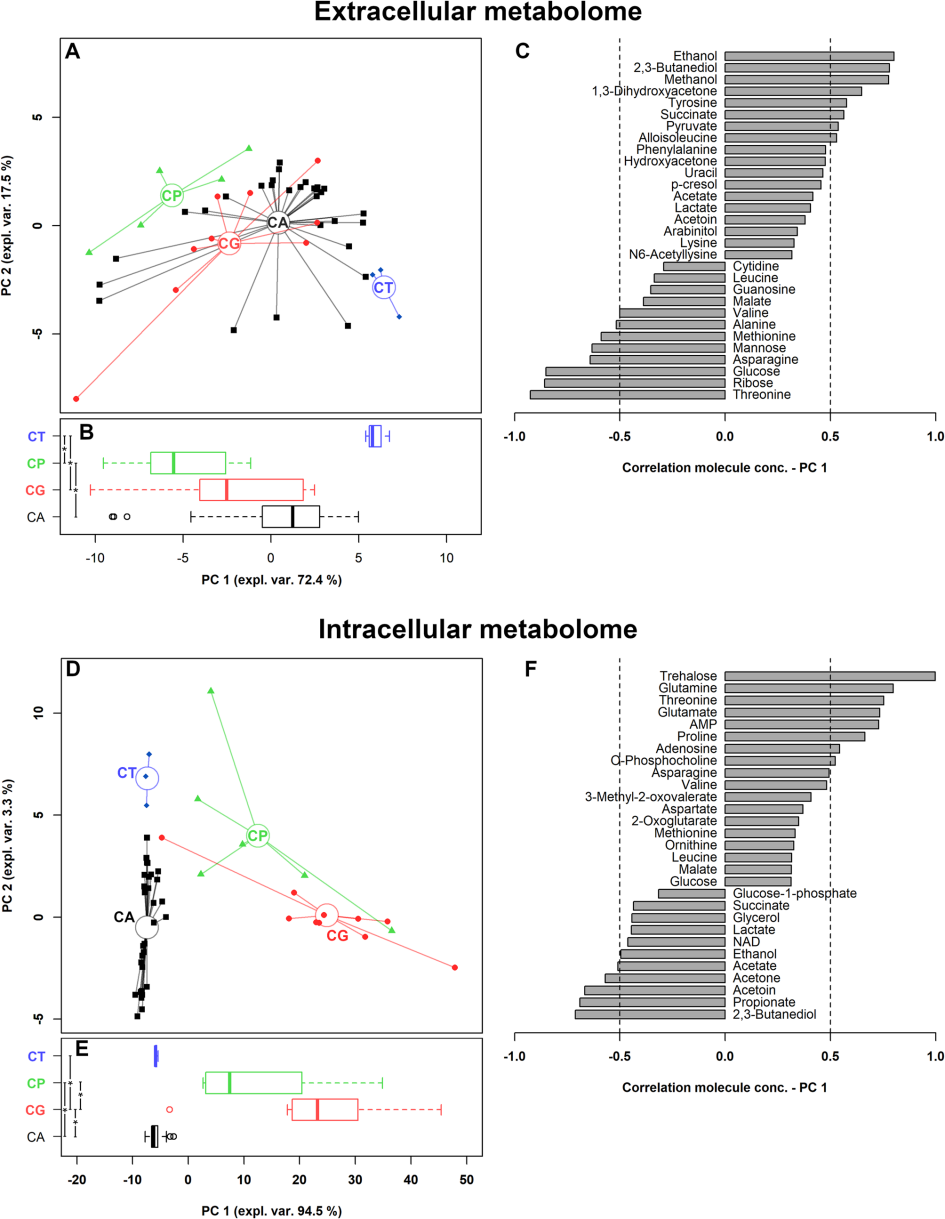
Correlation between extracellular and intracellular metabolome and *Candida* species. (**A**,**D**) Scoreplots of the rPCA models calculated on the spaces constituted by the concentration of the molecules significantly different among species. Empty circles highlight the median of the species. (**B**,**E**) Box plots summarizing the positions of the samples along PC 1. (**C**,**F**) Bar plots describing the correlation between the concentration of each molecule and its importance along PC 1. *CA* *C. albicans* complex, *CT* *C. tropicalis*, *CG* *C. glabrata*, *CP* *C. parapsilosis*. *P < 0.05.

We then evaluated whether *Candida* clinical isolates belonging to different complex/species showed differences in their metabolome profiles. Forty extracellular and 41 intracellular metabolites showed different concentrations when compared among the various complex/species (Supplementary Tables S4–S5). Twenty-one of them were in common between the two environments. The 40 and 41 molecules differing among *Candida* strains represented the 83% and 87% of all the molecules overall observed in the two environments. To grab the overall trends described by these molecules, two rPCA models were created, optimally described by 3 and 2 PCs, respectively (Fig. 4). In each case, PC 1 best described the peculiarities among the species, as it can be visually appreciated from the scoreplots in Fig. 4A,D. As it can be seen from the boxplots (Fig. 4B,E), in both cases *C. tropicalis* strains never showed statistically different PC 1 scores from *C. albicans* complex ones, in accordance with the dendrogram based on MALDI-TOF MS measurements (Fig. 1). Considering the extracellular metabolome profile, *C. albicans* complex and *C. tropicalis* strains, along with *C. glabrata*, were mainly characterized by higher concentrations of ethanol, 2,3-butanediol, tyrosine and succinate, and a more marked consumption of methanol, ribose, glucose and methionine, with respect to *C. parapsilosis* isolates (Fig. 4C). When the intracellular metabolome was considered (Fig. 4F), differences strictly mimicking those highlighted by MALDI-TOF MS could be observed, being *C. albicans* complex strains' PC 1 values similar to those of *C. tropicalis* group, and significantly different from *C. parapsilosis* and *C. glabrata* ones. In addition, *C. parapsilosis* and *C. glabrata* differed one from the other. Looking at intracellular metabolites, *C. albicans* complex and *C. tropicalis* strains showed high levels of 2,3-butanediol, propionate, acetoin and acetone; on the other side, *C. glabrata* isolates contained high concentrations of trehalose and the amino acids glutamine, threonine, glutamate and proline. The abundance of molecules with a concentration differing among the groups both in extracellular and intracellular metabolome did not allow to identify by ORA analysis a specific metabolic pathway mostly responsible for the sample’s distribution.

### Differential metabolites associated with *Candida albicans* sub-groups

**Figure 5:**
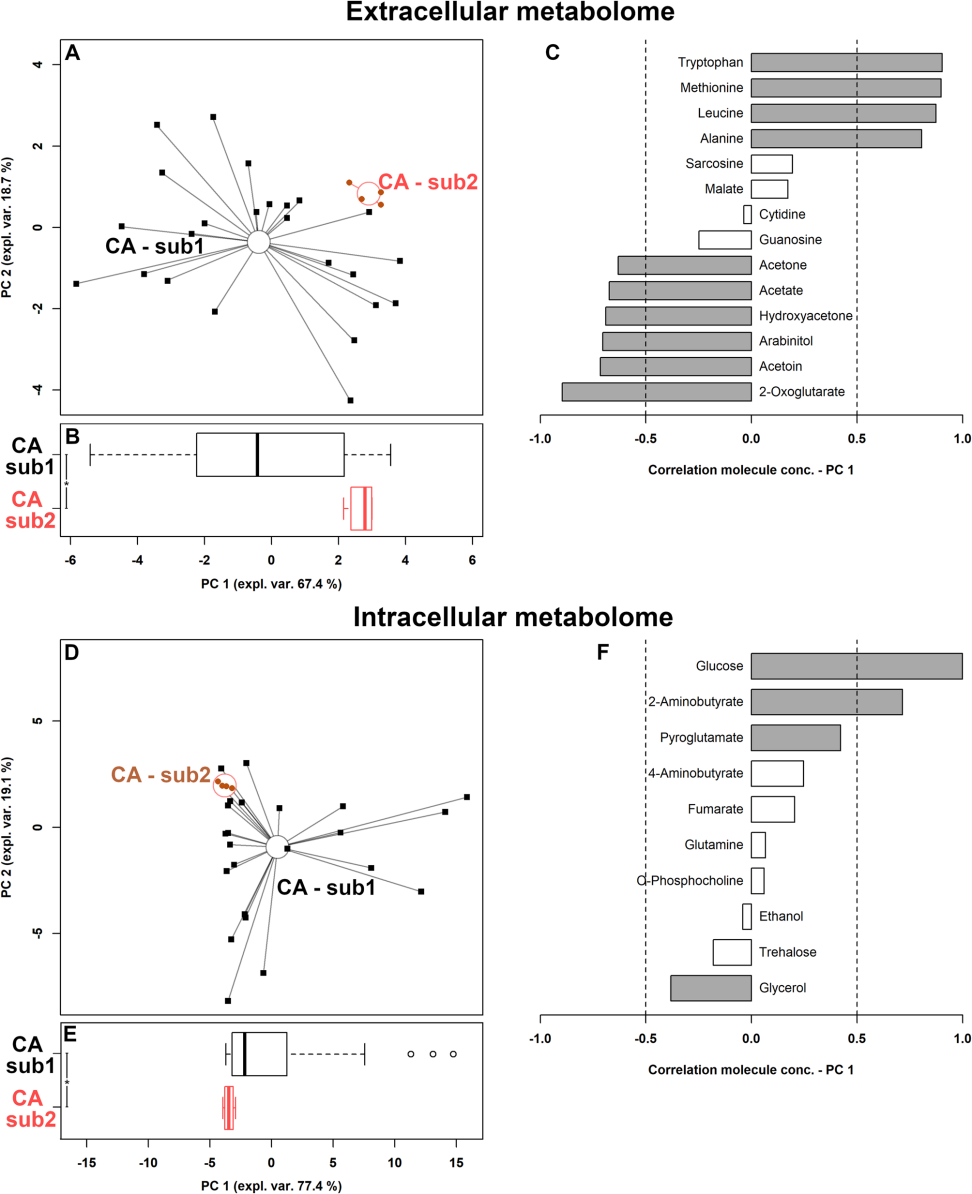
Correlation between extracellular and intracellular metabolome and *C. albicans* sub-groups. (**A**,**D**) rPCA models calculated on the spaces constituted by the concentration of the molecules significantly different between sub-groups. Empty circles highlight the median of the subgroup. (**B**,**E**) Box plots summarizing the positions of the samples along PC 1. (**C**,**F**) bar plots describing the correlation between the concentration of each molecule and its importance along PC 1. *CA* *C. albicans*. *P < 0.05.

To observe if the two *C. albicans* sub-groups visually identified by MALDI-TOF-based clustering corresponded to actual metabolomic differences, a univariate analysis was set up on a molecule-by-molecule basis. Fourteen molecules in the extracellular metabolome and 10 in the intracellular indeed differentiated the two sub-groups (Supplementary Tables S6–S7). The corresponding summarizing rPCA models were best described by 4 and 3 PCs, respectively (Fig. 5A,D). In both cases PC 1 optimally grabbed the differences between the sub-groups, so that their PC 1 scores appeared as statistically different (Fig. 5B,E). The discrepancies between the extracellular metabolome features of the two sub-groups revolved mainly around amino acids metabolism, with tryptophan, methionine, leucine and alanine mostly differentiating the two groups, together with 2-oxoglutarate (Fig. 5C). The intracellular molecule mostly contributing to the differentiation was glucose (Fig. 5F).

### Correlation between metabolome and virulence factors

We further investigated the correlations between the extracellular and intracellular metabolites and some important virulence-associated parameters for *Candida* pathogenesis, i.e. the ability to form biofilm, the resistance to different antifungal agents (caspofungin, fluconazole and amphotericin-B) and the production of SAP.

Firstly, we evaluated the capability of all *Candida* strains to form biofilm on an abiotic surface. In relation to this feature, we classified the strains into 4 categories, named 0 (no biofilm; n = 16), 1 (weak producers; n = 16), 2 (intermediate producers; n = 13), 3 (strong producers; n = 4) (Supplementary Table S1), and we searched for correlations with metabolites by means of the same methodological approach described in the previous paragraphs. Five extracellular and 9 intracellular metabolites showed significant different concentrations when compared among biofilm categories (Supplementary Tables S8–S9). Interestingly, the strongest biofilm producers were characterized by the highest concentrations of glutamate and lysine in the intracellular compartment.

**Figure 6:**
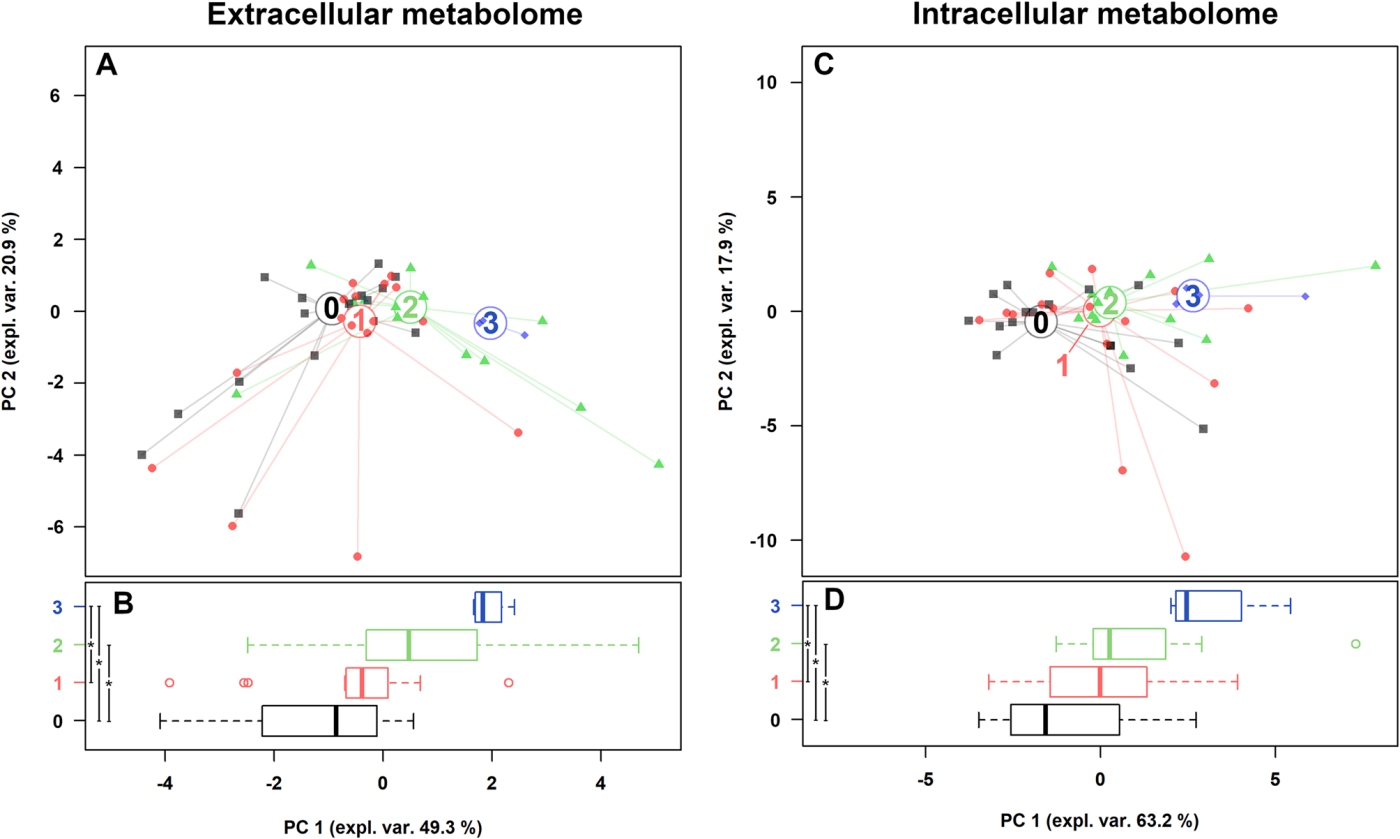
Correlation between extracellular and intracellular metabolome and the ability to form biofilm. (**A**,**C**) rPCA models calculated on the spaces constituted by the concentration of the molecules significantly different among *Candida* strains grouped on the basis of their ability to form biofilm (0: no biofilm; 1: weak producers; 2: intermediate producers; 3: strong producers). (**B**,**D**) Box plots summarizing the positions of the samples along PC 1. *P < 0.05.

rPCA models were able to separate *Candida* isolates on PC 1, as shown in Fig. 6A,C. Indeed, as pointed out in the boxplots (Fig. 6B,D), non-producing *Candida* strains (group 0) were statistically different from intermediate and strong biofilm producing strains (groups 2 and 3). Group 3 was also statistically differentiated from weak biofilm producers (group 1), for both extracellular and intracellular metabolome.

When analyzing the susceptibility to antifungal drugs (Supplementary Table S1), we found that all strains were susceptible to amphotericin-B with MIC values ranging between ≤ 0.007 and 0.125 µg/mL. A total of 17 strains (4 *C. albicans*, 1 *C. parapsilosis*, 2 *C. tropicalis*, and all 10 *C. glabrata* strains) were resistant to fluconazole, whereas 6 to caspofungin (1 *C. dubliniensis* and 5 *C. parapsilosis*). Interestingly, all *C. albicans* fluconazole-resistant strains were SAP producers (Supplementary Table S1), as previously reported^23^.

Twenty-two extracellular and 24 intracellular metabolites showed significant different concentrations between fluconazole-resistant and susceptible strains (Supplementary Tables S10–S11), as well as 19 extracellular and 16 intracellular molecules were significantly different based on caspofungin susceptibility (Supplementary Tables S12–S13).

It is worth of note that both fluconazole and caspofungin-resistant strains showed higher threalose concentrations in the intracellular compartment, compared to susceptible isolates.

**Figure 7:**
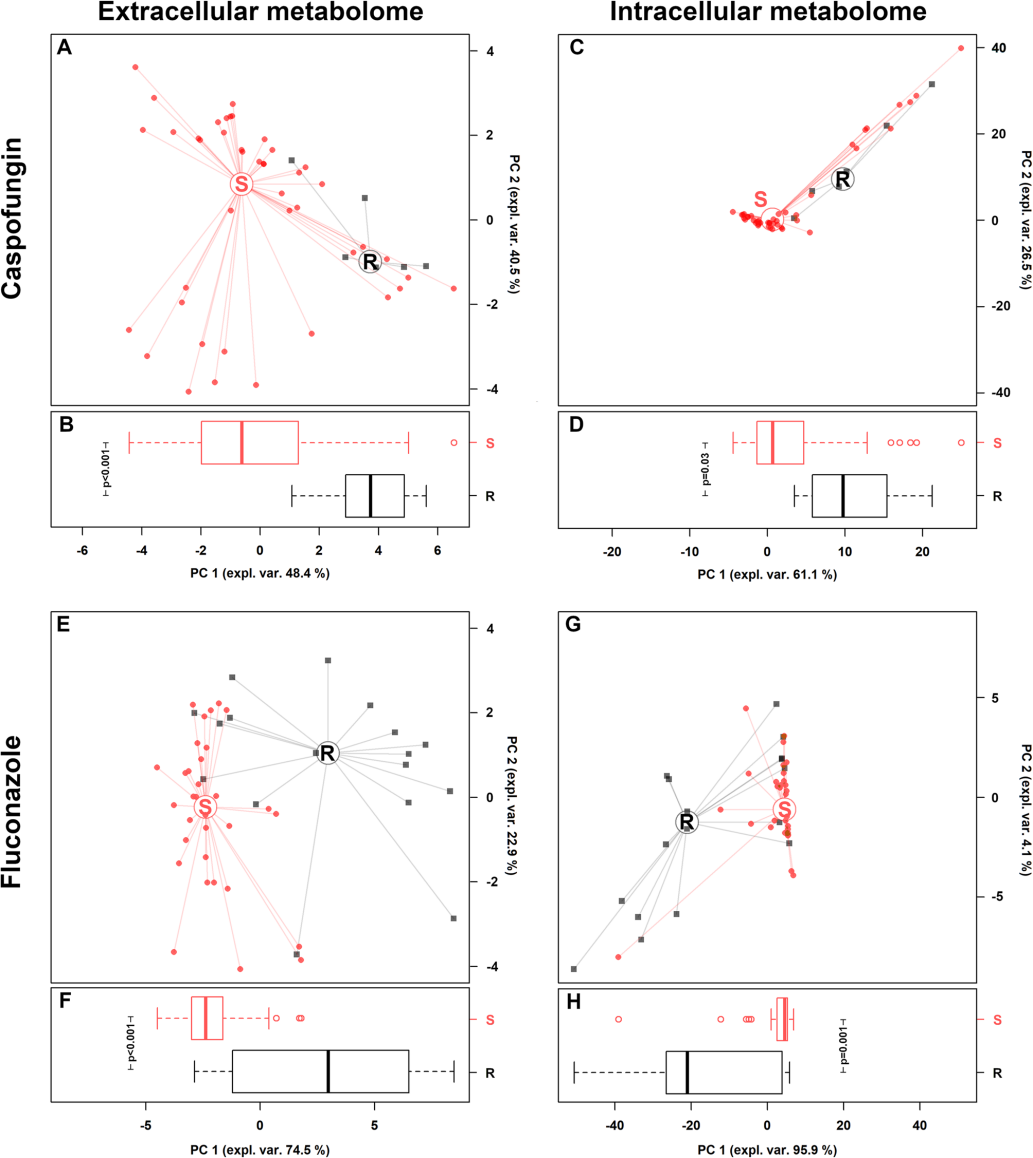
Correlation between extracellular and intracellular metabolome and *Candida* susceptibility to caspofungin and fluconazole. (**A**,**C**,**E**,**G**) rPCA models calculated on the spaces constituted by the concentration of the molecules significantly different between susceptible (S) and resistant (R) *Candida* strains. (**B**,**D**,**F**,**H**) Box plots summarizing the positions of the samples along PC 1. *P < 0.05.

rPCA models were able to separate *Candida* isolates on PC 1, as shown in Fig. 7A,C,E,G. Indeed, as pointed out in the boxplots (Fig. 7B,D,F,H), susceptible *Candida* strains (S) were statistically different from resistant ones (R), for both extracellular and intracellular metabolome.

Finally, *Candida* strains were categorized based on SAP activity (Supplementary Table S1): 31 strains were not-producers, whereas 18 showed SAP activity (visible clear zone from 1 to 12 mm). Interestingly, the strongest SAP producers were two *C. albicans* strains isolated from respiratory tract.

**Figure 8:**
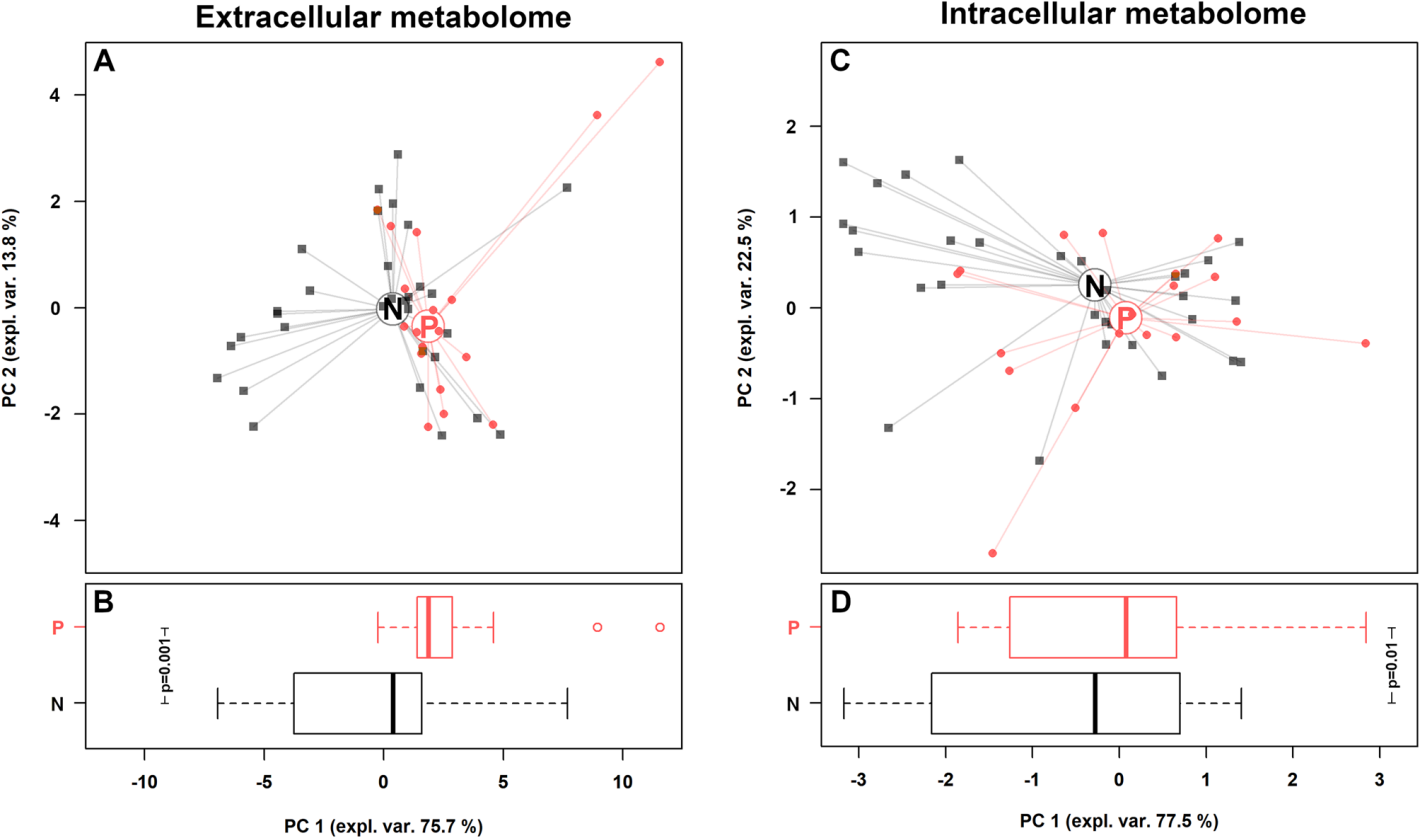
Correlation between extracellular and intracellular metabolome and SAP production. (**A**,**C**) rPCA models calculated on the spaces constituted by the concentration of the molecules significantly different between SAP producers (P) and non-producers (N). (**B**,**D**) Box plots summarizing the positions of the samples along PC 1. *P < 0.05.

Ten extracellular and 3 intracellular metabolites showed significant different concentrations between SAP-producers and non-producers (Supplementary Tables S14–S15). rPCA models were able to separate *Candida* isolates on PC 1, as shown in Fig. 8A,C. Indeed, as pointed out in the boxplots (Fig. 8B,D) SAP-producers (P) were statistically different from non-producers (N), for both extracellular and intracellular metabolome.

## Discussion

In recent years, methods for the rapid identification of microorganisms have been developed, which have enabled the detection of new species and characterization of their components and/or metabolites. These methods have been based on proteomic profiles, using MALDI-TOF MS, and metabolome profiles, by ^1^H-NMR and hyphenated mass spectrometry^8,12–15^.

*Candida albicans* is the most common cause of candidiasis, followed by *C. glabrata* and *C. parapsilosis*^9,24–26^. Invasive infections by these fungi have a high morbidity and mortality rate, so rapid identification and novel approaches for their characterization are of great interest to improve the diagnosis and the knowledge about their virulence and pathogenic properties.

Protein identification by MALDI-TOF MS may provide data for comparing *Candida* spp. virulence factors, such as phospholipases or aspartyl proteases^27,28^.

Metabolomic analysis by ^1^H-NMR is an intrinsically quantitative platform and therefore is extremely simple and quick^29^. In our work, this technique allowed to identify and quantify 66 metabolites produced by different *Candida* species, recovered from different sites of infection. These metabolites are associated with glycolysis or gluconeogenesis, tricarboxylic acid (TCA) cycle, nucleic acids synthesis and amino acids and lipids metabolism. These metabolic pathways are mainly related to energy production and also to virulence mechanisms^20,30–33^.

*Candida* clinical isolates recovered from blood, respiratory tract and vaginal environment showed differences in their metabolome profiles. The metabolites ethanol, 2,3-butanediol, hydroxyacetone, isobutyrate and 1,3-dihydroxyacetone showed different concentrations in the extracellular metabolome, when compared among isolates from different sources. These molecules are products of the glucose metabolism by *Candida* spp.^22,34^. *Candida* isolates from the respiratory tract showed high concentrations of ethanol in the extracellular metabolome. *Candida* spp. are relatively intolerant to ethanol and therefore secrete ethanol into the extracellular medium^34^. However, ethanol can be used as an alternative carbon source in the absence of glucose, which is converted into the central metabolite acetyl-CoA and used to produce glucose and energy^35,36^.

Further considering the extracellular metabolome, succinate and, to a lesser extent, citrate showed different concentrations among the groups analyzed. These molecules are also related to energy production, being involved in the tricarboxylic acid cycle^30^.

Nine *Candida* intracellular metabolites were significantly different in relation to the source of infection. These molecules are mainly involved in the metabolism of glucose (i.e. pyruvate, ADP, NAD, 2-aminobutyrate), amino acids (i.e. aspartate, lysine, creatinine) and nucleic acids (i.e. uracil and UMP)^20,32,37^. Blood *Candida* isolates showed higher concentrations of aminobutyrate than the others. Aminobutyrate is related to the gamma-aminobutyric acid (GABA) and it can be also used for succinate synthesis^38^. These metabolites can also be related to the virulence because GABA increases in vitro germ-tube formation and phospholipase B1 expression in *C. albicans*^39^.

*Candida* isolates from the respiratory tract showed higher concentrations of UMP and uracil. There may be subtle relationships between uridine uptake, cell adhesion, growth rate, the amount or composition of mannoproteins or other components of *C. albicans* cell wall^40^. UMP and uracil could be related to *Candida* virulence, as they are important for the pathogenicity of the opportunistic mould *Aspergillus fumigatus*^41^.

In this study, the highest concentrations of aspartate, lysine and creatinine were observed in respiratory tract, blood and vagina isolates, respectively. Aspartate is an amino acid associated with virulence and stress due to the exposure to antifungals^20^.

In addition, SAP are globular proteins with two aspartate catalytic sites, which are involved in the adhesion, tissue invasion and in the degradation of structural proteins of the host's immune system^42–44^. In our in vitro studies, the strongest SAP producers were strains isolated from the respiratory tract with no significant relation between SAP activity and higher concentrations of aspartate in the metabolome.

Another amino acid related to virulence and stress response is lysine: *C. albican*s increases lysine levels after exposure to ketoconazole^20^. Lysine acetylation is a prevalent modification in enzymes that catalyze intermediate metabolism and *C. glabrata* increases cellular lysine deacetylase activity in response to the intracellular milieu of macrophage^45,46^.

In relation to the taxonomy, 40 extracellular and 41 intracellular metabolites showed different concentrations when compared among *Candida* species. Differences strictly mimicking those highlighted by MALDI-TOF MS could be observed mainly in the intracellular metabolome. *C. albicans* complex and *C. tropicalis* strains showed high levels of 2,3-butanediol, propionate, acetoin and acetone. These metabolites are microbial fermentation products and can also be related to fungal virulence^47^. On the other hand, *C. glabrata* isolates contained high concentrations of trehalose and the amino acids glutamine, threonine, glutamate, and proline. In addition to serve as an alternative carbon source, trehalose has a critical role in the morphogenesis and response to fungal stress, especially dehydration, thermal and oxidative stress^48–50^. Amino acids are not only cell signaling molecules but are also important in fungal virulence as regulators of gene expression, morphogenesis and biofilm growth, key precursors for syntheses of hormones and low-molecular weight nitrogenous substances with enormous biological importance^51,52^. Previous studies also suggested the role of proline in reducing the production of reactive oxygen species in the mitochondria^53^.

Two *C. albicans* sub-groups were visually identified by MALDI-TOF-based clustering and showed metabolomic differences in the levels of intracellular glucose and extracellular amino acids (i.e. tryptophan, methionine, leucine, and alanine). These data, indeed, showed how the metabolomic analysis by ^1^H-NMR is effective at identifying *Candida* species and even subgroups. Over again, some amino acids contributed to the differentiation among the groups which are not only related to primary metabolism but also to fungal virulence^51^.

Moreover, we found specific metabolic fingerprints associated with different *Candida* virulence-associated features, such as the ability to form biofilm and the antifungal resistance.

In relation to biofilm formation, we confirmed the importance of amino acids in biofilm growth, as demonstrated by the higher concentrations of glutamate and lysine in the intracellular compartment of the strongest producer strains^51^.

Regarding the in vitro testing of antifungal drugs, we observed that both fluconazole and caspofungin-resistant strains showed higher threalose concentrations in the intracellular compartment. In this context, it has been shown that threalose biosynthesis pathway could be the target of new therapeutic drugs, opening new perspectives in the field of antifungal therapies^49^.

In conclusion, our data showed that *Candida* spp. produce different amounts of metabolites depending on the site of infection, the species and the virulence factors. Therefore, the metabolomic analyses can be used not only for identification of *Candida* species but also for pathogenicity studies.

Further studies, including a larger number of strains, are needed to better understand the metabolic pathways involved in pathogenic and biological-associated properties of *Candida* spp. Moreover additional experiments with more specialized media mimicking the different sources of infection, will shed light on the priming of fungal metabolism in the different ecological niches.

## Methods

### Strains and culture conditions

Forty-nine *Candida* strains were collected at the Bacteriology Laboratory, Sant’Orsola Malpighi University Hospital, Bologna, Italy, during the period October–November 2018 during routine diagnostic procedures. The 49 *Candida* strains were collected from respiratory tract, vaginal swabs, and blood, and were kept anonymous. Samples were seeded on Sabouraud dextrose agar (Vakutest Kima, Padova, Italy) and were grown aerobically at 37 °C. The identification at the species level was obtained by means of a matrix-assisted laser desorption/ionization time-of-flight mass spectrometry (MALDI-TOF MS), using Bruker instrument (Bruker Daltonics, Bremen, Germany)^12^.

### MALDI-TOF MS sample preparation and analysis

Sample preparation for MALDI-TOF MS analysis was performed as previously described by Foschi et al.^12^. Cell pellets corresponding to 10^8^ colony forming unit (CFU) (24-h cultures) were washed with 300 μL of sterile water and 900 μL of absolute ethanol, then suspended in 25 μL of 70% formic acid and 25 μL of pure acetonitrile. The solutions were thoroughly vortexed and centrifuged at 18,000×*g* for 10 min. Afterwards, 1 μL of the supernatants was spotted in six replicates on a ground-steel MALDI target plate (Bruker Daltonics), dried at room temperature and overlaid with 1 μL of MALDI HCCA matrix solution (10 mg/mL of α-ciano-4-hydroxycinnamic acid in 50% acetonitrile-2.5% trifluoroacetic acid; Bruker Daltonics). A MALDI-TOF MS measurement was performed using a Bruker instrument (Bruker Daltonics) operating in linear, positive ion mode and using the Flex Control 3.3 software with the following parameters: laser frequency: 20%; ion extraction delay time, 30 ns; ion source voltage one, 19 kV; ion source voltage two, 15.8 kV; and ion source lens voltage, 7.75 kV. A total of 240 laser shots was automatic acquired for each spectrum.

For species identification, spectra collected within a mass range of 2000–20,000 Da were analyzed with Bruker Biotyper 3.1 software and compared with the ones of the reference database.

A clustering analysis of all the *Candida* strains, belonging to different species, was performed by the generation of a score-oriented dendrogram. In particular, the MSPs of each strain were generated from at least 8 technical replicates (the ones with the highest score values at the species identification) using the MALDI Biotyper 3.1 software, with default setting parameters^12^. A peak quality control was performed using FlexAnalysis software 3.3 (Bruker Daltonics): spectra with outlier peaks or anomalies were removed from the spectra set of the *Candida* strain. The relationship between MSPs obtained from each strain was visualized in a score-oriented dendrogram using the average linkage algorithm implemented in the MALDI Biotyper 3.1 software. MSPs were created in relation to their mass signals and peak intensities.

### ITS sequencing

To confirm *Candida* identification at the species level, ITS sequencing was performed as follows. Total DNA of *Candida* isolates was extracted by Versant sample preparation module (Siemens Healthcare Diagnostics, Tarrytown, NY, USA) and amplified using ITS1/4 primers^54^. Amplicons were sequenced, edited using BioEdit Sequence Alignment program and compared with reference ITS regions deposited in the GenBank Database.

### *Candida* fractions preparation

*Candida* strains were grown in Sabouraud Dextrose Agar at 37 °C for 24 h. *Candida* suspensions were prepared in water to an optical density (OD 600 nm) of 0.1, corresponding to a cell concentration of 10^6^ CFU/mL. Aliquots of 0.5 mL of *Candida* suspensions were added to 5 mL of Sabouraud Dextrose Broth and the tubes were incubated at 37ºC and 180 rpm for 24 h. The turbidity of the *Candida* cultures was adjusted to an optical density (OD 600 nm) of 0.8. Cell suspensions were centrifuged at 5000×*g* for 5 min at 4 °C, then supernatants were filtered through a 0.22 μm membrane filter to obtain cell free supernatants and analyzed by ^1^H-NMR to examine the extracellular metabolome. Cell pellets were washed in sterile water, re-suspended in 500 μL of lysis buffer (20 mM Tris HCl pH 8, 2 mM sodium EDTA, 1.2% Triton X-100) and then vortexed with 0.2 g of glass beads to ensure a complete lysis. Glass beads were then precipitated by centrifugation (4700×*g* for 5 min) and the supernatants, containing metabolites, were collected, and employed for ^1^H-NMR analysis of the intracellular metabolome, as described below.

### Metabolomics investigation

Each cell free supernatant (700 µl) and lysate (500 µl) obtained from *Candida* cultures was added to 100 μL of a D_2_O solution of 3-(trimethylsilyl)-propionic-2,2,3,3-d4 acid sodium salt (TSP) 10 mM set to pH 7.0 by means of a 1 M phosphate buffer. An AVANCE III spectrometer (Bruker, Milan, Italy) was employed to register ^1^H-NMR spectra at 298 K and at a frequency of 600.13 MHz. Broad signals from slowly tumbling molecules were reduced with a T_2_ filter of 400 echoes, separated by an echo time of 400 µs. Signals assignment was performed by Chenomx (Chenomx Inc., Canada, ver 8.02). Spectra processing and molecules quantification was performed, by following Foschi et al.^55^, in R computational language^56^, while artwork was refined by GIMP (version 2.10, www.gimp.org). Prior to univariate analysis, data were transformed to normality by means of BoxCox transformation^57^. Differences between two groups were highlighted by t test, while differences among three or more groups were highlighted by ANOVA test followed by Tukey HSD test, by taking advantage of the corresponding functions implemented in the R package “agricolae”. For the above statistical tests, a cut-off *P* value of 0.05 was accepted. Metabolic pathway analysis was conducted though Reactome pathway knowledgebase^58^, with over representation analysis (ORA) based on hypergeometric test^59^.

### Biofilm formation assay

*Candida* suspensions were prepared in sterile water from 24 h Sabouraud Dextrose Agar cultures, to an OD 600 nm of 0.8–1.2 (corresponding to 5 × 10^6^–10^7^ CFU/mL). Water suspensions were diluted 1:10 in SDB and inoculated in a sterile 96-well flat-bottomed plastic plate (200 μL/well) and allow to grow for 24 h at 37 °C under gentle shaking conditions (50 rpm). Afterwards, the medium was removed and the adherent biofilms were stained by crystal violet^60^. Briefly, biofilms were gently washed with PBS twice, then fixed with 200 μL of 99% ethanol for 15 min, before being stained for 2 min with 1% (w/V) crystal violet in 12% ethanol. Excess stain was rinsed out by washing the multi-well plate with water for three times. Subsequently, the plate was air dried, the dye bound to the adherent *Candida* cells was re-solubilized in 200 μL of 33% (V/V) ethanol and the absorbance was measured at 595 nm using a EnSpire Multimode Plate Reader (Perkin-Elmer). All assays were performed in quadruplicate.

### Antifungal susceptibility testing

All *Candida* strains were tested for antifungal susceptibility by broth microdilution method, following EUCAST guidelines (version 7.3.2, available at: https://www.eucast.org/astoffungi/). In particular, caspofungin, fluconazole and amphotericin-B were tested and MIC values were interpreted by EUCAST clinical breakpoints.

### SAP production assay

All *Candida* isolates were tested in vitro fot the ability to produce SAP. Briefly, SAP activity was assessed by a culture-based method, using a medium composed as follows: 1.17% yeast carbon base (Sigma Aldrich, Milan, Italy), 0.01%; yeast extract (Sigma Aldrich), 0.2% bovine serum albumin (Sigma Aldrich). The medium was adjusted to pH 5, sterilized by filtration, and added to the stock solution of autoclaved 2% agar. Plates were incubated for 7 days at 37° C in aerobic condition. *Candida* strains were classified in non-producers (when no visible clarification of the agar around the colony was present) or SAP producers (when visible clear zone ≥ 1 mm was detected).

## Supplementary information

Supplementary Information

## Data Availability

Raw metabolomic data were deposited in the Metabolights repository (https://www.ebi.ac.uk/metabolights/), under the accession number MTBLS1978. ITS sequences of all *Candida* strains were deposited in GenBank under the accession numbers (MT876136-84) (Supplementary Table S1).
