# Supplementary material for: Metabolic profiling of Candida clinical isolates of different species and infection sources
Source: Sci Rep. 2020 Oct 7;10:16716. doi: 10.1038/s41598-020-73889-1 (PMC7541501; doi:10.1038/s41598-020-73889-1)
Supplement: Supplementary file 1 — Supplementary Information [file 41598_2020_73889_MOESM1_ESM.pdf]

Metabolic profiling of *Candida* clinical isolates of different species and infection sources

Josidel Conceição Oliver[1,4#], Luca Laghi[2#], Carola Parolin[1], Claudio Foschi[3], Antonella Marangoni[3], Andrea Liberatore[3], Amanda Latercia Tranches Dias[4], Monica Cricca[3]*, Beatrice Vitali[1]

[1]Department of Pharmacy and Biotechnology, Alma Mater Studiorum, University of Bologna, Bologna, Italy;

[2]Centre of Foodomics, Department of Agro-Food Science and Technology, Alma Mater Studiorum, University of Bologna, Cesena, Italy;

[3]Microbiology, DIMES, Alma Mater Studiorum, University of Bologna, Bologna, Italy;

[4]Department of Microbiology and Immunology, Federal University of Alfenas, Minas Gerais, Brazil.

Supplementary information

Table S1: Infection source and virulence factors of *Candida* strains.

Strain number	Acc. Num. (GenBank)	Species	Source	Biofilm	SAP activity (mm)	Amphoter-B	Fluconazole	Caspofungin
24	MT876150	*C. albicans*	R	0	N	S (0,03)	S (1)	S (0,015)
25	MT876145	*C. glabrata*	R	1	N	S (0,125)	R (>32)	S (0,0625)
26	MT876175	*C. albicans*	R	3	N	S (0,0625)	S (0,5)	S (0,015)
28	MT876158	*C. albicans*	R	1	P (12)	S (0,03)	R (>32)	S (0,015)
29	MT876184	*C. albicans*	R	0	P (7)	S (0,03)	R (>32)	S (0,015)
30	MT876144	*C. tropicalis*	R	3	N	S (0,125)	R (>32)	S (0,03)
31	MT876151	*C. parapsilosis*	B	2	P (2)	S (0,0625)	S (1)	I (0,125)
33	MT876147	*C. parapsilosis*	R	1	P (2)	S (0,0625)	I (4)	S (0,25)
44	MT876146	*C. glabrata*	R	1	N	S (0,0625)	R (>32)	S (0,0625)
46	MT876136	*C. glabrata*	R	0	N	S (0,125)	R (>32)	S (0,0625)
47	MT876153	*C. glabrata*	V	0	N	S (0,125)	R (>32)	S (0,03)
48	MT876152	*C. glabrata*	V	0	N	S (0,125)	R (>32)	S (0,03)
51	MT876138	*C. parapsilosis*	B	2	N	S (0,0625)	S (0,5)	S (0,25)
52	MT876148	*C. parapsilosis*	B	2	N	S (<=0,007)	S (<=0,25)	S (<=0,007)
53	MT876137	*C. parapsilosis*	B	3	P (5)	S (0,03)	S (2)	S (0,25)
59	MT876169	*C. albicans*	B	2	P (2)	S (0,03)	R (>32)	S (0,03)
60	MT876179	*C. albicans*	B	1	P (3)	S (0,03)	S (1)	S (0,03)
61	MT876163	*C. albicans*	B	1	N	S (0,03)	S (0,5)	S (0,03)
66	MT876170	*C. albicans*	B	1	N	S (0,03)	S (1)	S (0,03)
67	MT876154	*C. glabrata*	B	2	N	S (0,125)	R (>32)	S (0,0625)
71	MT876167	*C. albicans*	V	2	N	S (<=0,007)	S (<=0,25)	S (<=0,007)
72	MT876161	*C. albicans*	V	1	N	S (0,03)	S (0,5)	S (0,015)
73	MT876140	*C. glabrata*	V	1	N	S (0,0625)	R (>32)	S (0,0625)
74	MT876139	*C. glabrata*	V	0	N	S (0,03)	R (>32)	S (0,0625)
75	MT876143	*C. glabrata*	V	1	N	S (0,125)	R (>32)	S (0,0625)
76	MT876142	*C. glabrata*	V	0	N	S (0,25)	R (>32)	S (0,0625)
77	MT876168	*C. albicans*	V	1	P (3)	S (0,03)	S (1)	S (0,015)
78	MT876141	*C. albicans*	V	0	P (5)	S (0,0625)	S (0,5)	S (0,015)
79	MT876176	*C. albicans*	V	0	N	S (0,0625)	S (0,5)	S (0,015)
80	MT876173	*C. albicans*	B	0	P (2)	S (0,03)	S (0,5)	S (0,015)
81	MT876178	*C. albicans*	B	0	N	S (0,0625)	S (1)	S (0,015)
83	MT876156	*C. parapsilosis*	B	2	P (2)	S (0,0625)	S (1)	S (0,25)
85	MT876165	*C. albicans*	V	1	N	S (0,03)	S (0,5)	S (0,015)
86	MT876159	*C. albicans*	R	1	N	S (0,03)	S (0,5)	S (0,015)
87	MT876166	*C. albicans*	V	0	N	S (0,03)	S (0,5)	S (0,015)
88	MT876160	*C. albicans*	V	1	N	S (0,0625)	S (0,5)	S (0,015)
89	MT876183	*C. albicans*	V	0	P (5)	S (0,0625)	R (>32)	S (0,015)
91	MT876162	*C. dubliniensis*	R	1	N	S (0,015)	S (<=0,25)	R (>1)
92	MT876149	*C. albicans*	R	0	N	S (0,03)	S (0,5)	S (0,03)
93	MT876172	*C. albicans*	R	1	N	S (0,0625)	S (0,5)	S (0,03)
94	MT876174	*C. albicans*	V	0	P (1)	S (0,03)	S (0,5)	S (0,03)
95	MT876164	*C. albicans*	V	0	N	S (0,03)	S (0,5)	S (0,03)
96	MT876181	*C. albicans*	V	2	N	S (0,03)	S (1)	S (0,015)
97	MT876180	*C. albicans*	R	2	P (3)	S (0,03)	S (1)	S (0,015)
98	MT876177	*C. albicans*	B	2	P (4)	S (0,03)	S (1)	S (0,015)
99	MT876171	*C. albicans*	B	2	P (1)	S (0,03)	S (1)	S (0,015)
100	MT876155	*C. tropicalis*	R	3	P (5)	S (0,125)	R (>32)	S (0,03)
103	MT876157	*C. tropicalis*	B	2	P (5)	S (<=0,007)	S (1)	S (<=0,007)
104	MT876182	*C. albicans*	R	2	N	S (0,0625)	S (2)	S (0,03)

Source: R=RESPIRATORY SAMPLES; B=BLOOD SAMPLES; V=VAGINAL SWAB
Biofilm formation: 0=NON PRODUCER; 1=WEAK PRODUCER; 2=INTERMEDIATE PRODUCER; 3=STRONG PRODUCER. The activity was measured as absorbance at 595 nm.
SAP activity: N=NON-PRODUCER; P=PRODUCER; In brackets diameter of the visible clear zone around the colony after 1 week of incubation at 37°C in humidified chamber.
Antifungal susceptibility testing (Amphotericin-B, Fluconazole and Caspofungin): S=SUSCEPTIBLE; I=INTERMEDIATE; R=RESISTANT (interpretation following EUCAST antifungal clinical breakpoints); In brackets MIC values.

Table S2: Concentration (mmol/L, mean ± sd) of *Candida* extracellular metabolites significantly different in relation to the source of infection, expressed as difference from growth medium.

	Respiratory tract	Vagina	Blood
Inosine	$1.58x10^{-3} \pm 2.42x10^{-3}$ [ab]	$2.58x10^{-3} \pm 3.80x10^{-3}$ [a]	$5.47x10^{-5} \pm 1.76x10^{-3}$ [b *]
1,3-Dihydroxyacetone	$1.62x10^{-3} \pm 4.34x10^{-4}$ [a]	$1.08x10^{-3} \pm 7.11x10^{-4}$ [b]	$1.21x10^{-3} \pm 7.34x10^{-4}$ [ab]
Hydroxyacetone	$5.88x10^{-3} \pm 2.83x10^{-3}$ [a]	$4.47x10^{-3} \pm 2.46x10^{-3}$ [ab]	$2.55x10^{-3} \pm 2.52x10^{-3}$ [b]
Choline	$-1.62x10^{-2} \pm 2.11x10^{-2}$ [ab]	$-8.68x10^{-3} \pm 2.09x10^{-2}$ [a]	$-2.11x10^{-2} \pm 1.10x10^{-2}$ [b]
Putrescine	$-2.52x10^{-3} \pm 5.92x10^{-3}$ [ab]	$-3.71x10^{-3} \pm 6.82x10^{-3}$ [b]	$4.00x10^{-3} \pm 1.15x10^{-2}$ [a]
Lysine	$1.08x10^{-1} \pm 5.24x10^{-2}$ [ab]	$1.33x10^{-1} \pm 3.94x10^{-2}$ [a]	$7.37x10^{-2} \pm 7.44x10^{-2}$ [b]
Citrate	$3.47x10^{-1} \pm 1.24x10^{-1}$ [ab]	$4.09x10^{-1} \pm 5.87x10^{-2}$ [a]	$3.22x10^{-1} \pm 9.13x10^{-2}$ [b]
Succinate	$5.29x10^{-1} \pm 1.78x10^{-1}$ [a]	$4.15x10^{-1} \pm 1.87x10^{-1}$ [ab]	$2.92x10^{-1} \pm 2.16x10^{-1}$ [b]
Alloisoleucine	$5.14x10^{-1} \pm 1.63x10^{-1}$ [a]	$4.78x10^{-1} \pm 2.51x10^{-1}$ [ab]	$3.50x10^{-1} \pm 1.92x10^{-1}$ [b]
Ethanol	92.3 ± 21.9 [a]	87.8 ± 24.3 [ab]	70.2 ± 33.2 [b]
2,3-Butanediol	$2.64x10^{-1} \pm 1.45x10^{-1}$ [a]	$1.78x10^{-1} \pm 7.80x10^{-2}$ [ab]	$1.68x10^{-1} \pm 1.86x10^{-1}$ [b]
Isobutyrate	$1.62x10^{-2} \pm 9.45x10^{-3}$ [a]	$6.71x10^{-3} \pm 6.10x10^{-3}$ [b]	$1.01x10^{-2} \pm 1.08x10^{-2}$ [ab]

[*] Compact letters display of the comparisons between each pair of groups, where two mean values followed by a common superscript are not significantly different.

Table S3: Concentration (mmol/L, mean ± sd) of *Candida* intracellular metabolites significantly different in relation to the source of infection.

	Respiratory tract	Vagina	Blood
ADP	$2.77x10^{-3} \pm 9.97\ x10^{-4}$ [a]	$1.89x10^{-3} \pm 5.46\ x10^{-4}$ [b]	$2.62x10^{-3} \pm 8.93\ x10^{-4}$ [a *]
NAD	$6.95x10^{-3} \pm 1.10\ x10^{-3}$ [a]	$5.69x10^{-3} \pm 1.53\ x10^{-3}$ [b]	$6.44x10^{-3} \pm 1.58\ x10^{-3}$ [ab]
UMP	$1.22x10^{-2} \pm 2.88\ x10^{-3}$ [a]	$8.40x10^{-3} \pm 2.48\ x10^{-3}$ [b]	$1.09x10^{-2} \pm 3.03\ x10^{-3}$ [a]
Uracil	$7.97x10^{-3} \pm 1.93\ x10^{-3}$ [a]	$6.16x10^{-3} \pm 1.42\ x10^{-3}$ [b]	$6.76x10^{-3} \pm 1.90\ x10^{-3}$ [ab]
Creatinine	$5.72x10^{-3} \pm 2.57\ x10^{-3}$ [ab]	$7.29x10^{-3} \pm 3.58\ x10^{-3}$ [a]	$4.64x10^{-3} \pm 2.21\ x10^{-3}$ [b]
Lysine	$4.86x10^{-2} \pm 2.05\ x10^{-2}$ [ab]	$3.41x10^{-2} \pm 1.16\ x10^{-2}$ [b]	$6.06x10^{-2} \pm 4.78\ x10^{-2}$ [a]
Aspartate	$5.43x10^{-2} \pm 2.13\ x10^{-2}$ [a]	$3.45x10^{-2} \pm 1.86\ x10^{-2}$ [b]	$4.07x10^{-2} \pm 2.13\ x10^{-2}$ [ab]
Pyruvate	$1.91x10^{-2} \pm 1.01\ x10^{-2}$ [a]	$2.90x10^{-2} \pm 1.17\ x10^{-2}$ [b]	$2.01x10^{-2} \pm 1.33\ x10^{-2}$ [a]
2-Aminobutyrate	$4.86x10^{-3} \pm 1.47\ x10^{-3}$ [ab]	$4.50x10^{-3} \pm 1.06\ x10^{-3}$ [b]	$5.61x10^{-3} \pm 1.52\ x10^{-3}$ [a]

[*] Compact letters display of the comparisons between each pair of groups, where two mean values followed by a common superscript are not significantly different.

Table S4: Concentration (mmol/L, mean ± sd) of extracellular metabolites significantly different in relation to *Candida* species, expressed as difference from growth medium.

	C. albicans complex	*C. glabrata*	*C. parapsilosis*	*C. tropicalis*
Phenylalanine	$6.15x10^{-2} \pm 2.43x10^{-2}$ [a *]	$-7.18x10^{-3} \pm 3.67x10^{-2}$ [bc]	$-3.63x10^{-2} \pm 4.10x10^{-2}$ [c]	$3.62x10^{-2} \pm 1.82x10^{-2}$ [ab]
Tyrosine	$3.01x10^{-2} \pm 7.80x10^{-3}$ [b]	$4.57x10^{-2} \pm 1.70x10^{-2}$ [a]	$2.25x10^{-2} \pm 8.14x10^{-3}$ [b]	$3.88x10^{-2} \pm 6.31x10^{-3}$ [ab]
Fumarate	$3.34x10^{-2} \pm 1.39x10^{-2}$ [a]	$1.18x10^{-2} \pm 4.53x10^{-3}$ [b]	$8.29x10^{-3} \pm 2.76x10^{-3}$ [b]	$1.43x10^{-2} \pm 5.05x10^{-3}$ [b]
Orotate	$1.36x10^{-3} \pm 6.07x10^{-4}$ [ab]	$1.91x10^{-3} \pm 6.47x10^{-4}$ [a]	$1.22x10^{-3} \pm 7.66x10^{-4}$ [ab]	$7.68x10^{-4} \pm 7.04x10^{-4}$ [b]
Inosine	$3.73x10^{-4} \pm 1.36x10^{-3}$ [b]	$6.40x10^{-3} \pm 2.55x10^{-3}$ [a]	$-2.93x10^{-4} \pm 1.30x10^{-3}$ [b]	$-2.45x10^{-4} \pm 8.16x10^{-4}$ [b]
Cytidine	$-1.06x10^{-3} \pm 1.57x10^{-3}$ [b]	$4.78x10^{-3} \pm 2.40x10^{-3}$ [a]	$3.12x10^{-3} \pm 2.07x10^{-3}$ [a]	$-2.84x10^{-3} \pm 8.72x10^{-5}$ [b]
Guanosine	$-1.77x10^{-3} \pm 1.26x10^{-3}$ [b]	$3.62x10^{-3} \pm 1.63x10^{-3}$ [a]	$1.92x10^{-3} \pm 1.36x10^{-3}$ [a]	$-3.53x10^{-3} \pm 1.30x10^{-3}$ [b]
Uracil	$5.66x10^{-3} \pm 2.19x10^{-3}$ [b]	$-8.81x10^{-4} \pm 1.55x10^{-3}$ [c]	$3.97x10^{-3} \pm 2.39x10^{-3}$ [b]	$3.77x10^{-2} \pm 7.51x10^{-3}$ [a]
Sucrose	$1.48x10^{-2} \pm 3.69x10^{-3}$ [b]	$2.14x10^{-2} \pm 3.80x10^{-3}$ [a]	$1.55x10^{-2} \pm 4.24x10^{-3}$ [b]	$-2.51x10^{-2} \pm 1.50x10^{-2}$ [c]

Ribose	$-6.65x10^{-2} \pm 2.22x10^{-2}$ ^{b}	$-7.65x10^{-2} \pm 1.80x10^{-2}$ ^{b}	$-3.40x10^{-2} \pm 2.03x10^{-2}$ ^{a}	$-9.61x10^{-2} \pm 1.65x10^{-3}$ ^{c}
Mannose	$2.04x10^{-2} \pm 1.67x10^{-2}$ ^{b}	$3.09x10^{-3} \pm 2.16x10^{-2}$ ^{c}	$6.33x10^{-2} \pm 1.83x10^{-2}$ ^{a}	$-1.28x10^{-2} \pm 9.88x10^{-3}$ ^{c}
1,3-Dihydroxyacetone	$1.34x10^{-3} \pm 6.26x10^{-4}$ ^{ab}	$9.86x10^{-4} \pm 6.68x10^{-4}$ ^{b}	$1.19x10^{-3} \pm 7.52x10^{-4}$ ^{ab}	$2.17x10^{-3} \pm 1.43x10^{-4}$ ^{a}
Hydroxyacetone	$4.30x10^{-3} \pm 2.16x10^{-3}$ ^{ab}	$6.39x10^{-3} \pm 3.54x10^{-3}$ ^{a}	$8.63x10^{-4} \pm 1.50x10^{-3}$ ^{b}	$4.94x10^{-3} \pm 3.39x10^{-3}$ ^{ab}
Threonine	$-4.38x10^{-1} \pm 5.81x10^{-2}$ ^{ab}	$-3.99x10^{-1} \pm 7.82x10^{-2}$ ^{ab}	$-3.74x10^{-1} \pm 5.89x10^{-2}$ ^{a}	$-4.74x10^{-1} \pm 2.00x10^{-3}$ ^{b}
Lactate	$6.56x10^{-2} \pm 7.36x10^{-2}$ ^{a}	$6.38x10^{-3} \pm 3.40x10^{-2}$ ^{ab}	$7.74x10^{-2} \pm 6.79x10^{-2}$ ^{a}	$1.62x10^{-2} \pm 1.54x10^{-2}$ ^{ab}
Arabinitol	$1.85x10^{+0} \pm 1.13x10^{+0}$ ^{a}	$-3.83x10^{-2} \pm 2.98x10^{-2}$ ^{b}	$2.61x10^{+0} \pm 1.52x10^{+0}$ ^{a}	$1.21x10^{+0} \pm 7.46x10^{-1}$ ^{a}
Methanol	$-5.15x10^{-3} \pm 3.64x10^{-3}$ ^{a}	$-3.79x10^{-3} \pm 2.98x10^{-3}$ ^{a}	$-1.01x10^{-2} \pm 1.02x10^{-3}$ ^{b}	$-2.74x10^{-3} \pm 2.35x10^{-3}$ ^{a}
Glucose	$-3.28x10^{+1} \pm 9.90x10^{+0}$ ^{ab}	$-3.77x10^{+1} \pm 8.34x10^{+0}$ ^{bc}	$-1.92x10^{+1} \pm 9.73x10^{+0}$ ^{a}	$-4.57x10^{+1} \pm 5.77x10^{-2}$ ^{c}
Choline	$-2.11x10^{-2} \pm 6.70x10^{-3}$ ^{b}	$1.56x10^{-2} \pm 2.02x10^{-2}$ ^{a}	$-2.96x10^{-2} \pm 1.25x10^{-3}$ ^{c}	$-2.55x10^{-2} \pm 4.36x10^{-4}$ ^{bc}
Putrescine	$-1.24x10^{-3} \pm 9.69x10^{-3}$ ^{ab}	$-5.48x10^{-3} \pm 6.45x10^{-3}$ ^{b}	$6.12x10^{-3} \pm 3.19x10^{-3}$ ^{a}	$2.73x10^{-3} \pm 2.86x10^{-3}$ ^{ab}
Lysine	$1.32x10^{-1} \pm 3.71x10^{-2}$ ^{a}	$1.08x10^{-1} \pm 2.87x10^{-2}$ ^{a}	$-7.73x10^{-3} \pm 7.00x10^{-2}$ ^{b}	$7.49x10^{-2} \pm 2.35x10^{-2}$ ^{a}
Asparagine	$-1.64x10^{-1} \pm 2.16x10^{-2}$ ^{b}	$-1.15x10^{-1} \pm 3.85x10^{-2}$ ^{a}	$-1.62x10^{-1} \pm 1.01x10^{-2}$ ^{b}	$-1.73x10^{-1} \pm 5.20x10^{-3}$ ^{b}
Sarcosine	$1.17x10^{-3} \pm 3.69x10^{-3}$ ^{a}	$-1.05x10^{-2} \pm 3.79x10^{-3}$ ^{b}	$5.46x10^{-4} \pm 2.95x10^{-3}$ ^{a}	$-3.23x10^{-3} \pm 4.83x10^{-3}$ ^{a}
Malate	$-1.52x10^{-2} \pm 2.62x10^{-2}$ ^{b}	$3.33x10^{-2} \pm 2.85x10^{-2}$ ^{a}	$-3.81x10^{-2} \pm 2.32x10^{-2}$ ^{bc}	$-4.78x10^{-2} \pm 2.06x10^{-2}$ ^{c}
Succinate	$3.95x10^{-1} \pm 1.48x10^{-1}$ ^{b}	$5.65x10^{-1} \pm 1.68x10^{-1}$ ^{a}	$8.81x10^{-2} \pm 3.38x10^{-2}$ ^{c}	$7.67x10^{-1} \pm 6.25x10^{-2}$ ^{a}
Pyruvate	$6.20x10^{+0} \pm 2.88x10^{+0}$ ^{a}	$7.06x10^{+0} \pm 2.42x10^{+0}$ ^{a}	$1.02x10^{+0} \pm 6.19x10^{-1}$ ^{b}	$1.22x10^{+0} \pm 7.88x10^{-1}$ ^{b}
p-cresol	$2.70x10^{-3} \pm 2.75x10^{-3}$ ^{b}	$2.94x10^{-3} \pm 1.86x10^{-3}$ ^{ab}	$6.58x10^{-3} \pm 4.47x10^{-3}$ ^{ab}	$8.52x10^{-3} \pm 4.12x10^{-3}$ ^{a}
Acetone	$-3.34x10^{-3} \pm 2.43x10^{-3}$ ^{b}	$-4.82x10^{-4} \pm 2.67x10^{-3}$ ^{a}	$-1.99x10^{-3} \pm 3.01x10^{-3}$ ^{ab}	$-1.46x10^{-3} \pm 3.38x10^{-3}$ ^{ab}
Methionine	$-6.37x10^{-2} \pm 3.73x10^{-2}$ ^{a}	$-1.22x10^{-1} \pm 3.30x10^{-2}$ ^{b}	$-2.18x10^{-2} \pm 1.39x10^{-2}$ ^{a}	$-2.13x10^{-1} \pm 2.43x10^{-2}$ ^{c}
Acetate	$6.62x10^{-1} \pm 5.73x10^{-1}$ ^{a}	$-1.55x10^{-1} \pm 7.07x10^{-2}$ ^{b}	$1.61x10^{-1} \pm 5.34x10^{-1}$ ^{b}	$1.99x10^{-1} \pm 4.54x10^{-1}$ ^{ab}
N6-Acetyllysine	$-4.86x10^{-2} \pm 1.84x10^{-2}$ ^{b}	$-7.83x10^{-2} \pm 5.49x10^{-2}$ ^{c}	$-5.23x10^{-3} \pm 5.26x10^{-2}$ ^{b}	$1.01x10^{-1} \pm 4.07x10^{-2}$ ^{a}
Alanine	$1.30x10^{-2} \pm 9.93x10^{-2}$ ^{c}	$1.58x10^{-1} \pm 8.11x10^{-2}$ ^{b}	$4.72x10^{-1} \pm 2.64x10^{-1}$ ^{a}	$-3.87x10^{-1} \pm 2.47x10^{-2}$ ^{d}
Alloisoleucine	$3.91x10^{-1} \pm 1.54x10^{-1}$ ^{bc}	$7.10x10^{-1} \pm 1.82x10^{-1}$ ^{a}	$2.46x10^{-1} \pm 1.47x10^{-1}$ ^{c}	$5.93x10^{-1} \pm 1.18x10^{-1}$ ^{ab}
Acetoin	$1.67x10^{-1} \pm 8.59x10^{-2}$ ^{ab}	$5.89x10^{-2} \pm 3.27x10^{-2}$ ^{b}	$3.13x10^{-1} \pm 2.99x10^{-1}$ ^{a}	$2.03x10^{-1} \pm 4.45x10^{-2}$ ^{ab}
Ethanol	$8.29x10^{+1} \pm 2.14x10^{+1}$ ^{a}	$1.02x10^{+2} \pm 2.08x10^{+1}$ ^{a}	$4.23x10^{+1} \pm 2.28x10^{+1}$ ^{b}	$1.17x10^{+2} \pm 7.55x10^{+0}$ ^{a}
2,3-Butanediol	$2.01x10^{-1} \pm 9.03x10^{-2}$ ^{b}	$1.76x10^{-1} \pm 3.92x10^{-2}$ ^{b}	$4.25x10^{-2} \pm 4.03x10^{-2}$ ^{c}	$6.33x10^{-1} \pm 8.61x10^{-2}$ ^{a}
Isoleucine	$-3.21x10^{-2} \pm 1.51x10^{-2}$ ^{a}	$-7.51x10^{-2} \pm 1.46x10^{-2}$ ^{b}	$-5.09x10^{-2} \pm 1.89x10^{-2}$ ^{a}	$-1.00x10^{-1} \pm 2.38x10^{-2}$ ^{b}
Valine	$-2.58x10^{-2} \pm 2.10x10^{-2}$ ^{b}	$4.18x10^{-2} \pm 4.16x10^{-2}$ ^{a}	$-1.50x10^{-2} \pm 1.48x10^{-2}$ ^{b}	$-1.39x10^{-1} \pm 2.95x10^{-2}$ ^{c}
Leucine	$-7.97x10^{-3} \pm 1.09x10^{-1}$ ^{a}	$-1.58x10^{-1} \pm 1.24x10^{-1}$ ^{b}	$-8.72x10^{-2} \pm 8.74x10^{-2}$ ^{ab}	$-1.40x10^{-1} \pm 2.49x10^{-2}$ ^{ab}
2-Oxoisocaproate	$2.35x10^{-2} \pm 1.84x10^{-2}$ ^{c}	$3.89x10^{-2} \pm 3.88x10^{-2}$ ^{bc}	$1.17x10^{-1} \pm 4.29x10^{-2}$ ^{a}	$6.85x10^{-2} \pm 1.49x10^{-2}$ ^{ab}

[*] Compact letters display of the comparisons between each pair of groups, where two mean values followed by a common superscript are not significantly different.

Table S5: Concentration (mmol/L, mean ± sd) of intracellular metabolites significantly different in relation to *Candida* species.

	C. albicans complex	*C. glabrata*	*C. parapsilosis*	*C. tropicalis*
ADP	$2.48x10^{-3} \pm 8.27x10^{-4}$ ^{ab} [*]	$1.81x10^{-3} \pm 5.63x10^{-4}$ ^{b}	$2.28x10^{-3} \pm 1.02x10^{-3}$ ^{ab}	$3.82x10^{-3} \pm 6.97x10^{-4}$ ^{a}
Tyrosine	$9.88x10^{-3} \pm 2.32x10^{-3}$ ^{c}	$1.33x10^{-2} \pm 1.33x10^{-3}$ ^{b}	$9.94x10^{-3} \pm 1.96x10^{-3}$ ^{c}	$2.17x10^{-2} \pm 1.28x10^{-3}$ ^{a}
Fumarate	$3.65x10^{-3} \pm 5.74x10^{-4}$ ^{b}	$3.99x10^{-3} \pm 6.99x10^{-4}$ ^{b}	$3.21x10^{-3} \pm 8.01x10^{-4}$ ^{b}	$5.17x10^{-3} \pm 7.46x10^{-4}$ ^{a}
AMP	$1.14x10^{-2} \pm 3.27x10^{-3}$ ^{b}	$2.00x10^{-2} \pm 3.88x10^{-3}$ ^{a}	$1.83x10^{-2} \pm 4.19x10^{-3}$ ^{a}	$1.67x10^{-2} \pm 4.60x10^{-3}$ ^{ab}
Adenosine	$8.99x10^{-3} \pm 2.30x10^{-3}$ ^{b}	$1.24x10^{-2} \pm 3.20x10^{-3}$ ^{a}	$1.11x10^{-2} \pm 2.97x10^{-3}$ ^{ab}	$8.71x10^{-3} \pm 1.30x10^{-3}$ ^{b}
NAD	$6.90x10^{-3} \pm 1.33x10^{-3}$ ^{a}	$4.77x10^{-3} \pm 1.28x10^{-3}$ ^{b}	$6.07x10^{-3} \pm 1.01x10^{-3}$ ^{ab}	$6.38x10^{-3} \pm 7.71x10^{-4}$ ^{ab}
UMP	$9.40x10^{-3} \pm 2.98x10^{-3}$ ^{c}	$1.03x10^{-2} \pm 1.30x10^{-3}$ ^{bc}	$1.31x10^{-2} \pm 1.92x10^{-3}$ ^{ab}	$1.54x10^{-2} \pm 4.79x10^{-3}$ ^{a}
Glucose-1-phosphate	$3.90x10^{-2} \pm 2.53x10^{-2}$ ^{a}	$1.19x10^{-2} \pm 5.37x10^{-3}$ ^{b}	$2.76x10^{-2} \pm 1.53x10^{-2}$ ^{ab}	$1.66x10^{-2} \pm 1.08x10^{-2}$ ^{ab}
Sucrose	$1.22x10^{-2} \pm 4.97x10^{-3}$ ^{ab}	$1.69x10^{-2} \pm 1.29x10^{-2}$ ^{a}	$6.32x10^{-3} \pm 2.39x10^{-3}$ ^{b}	$6.57x10^{-3} \pm 6.83x10^{-4}$ ^{ab}
Threonine	$1.25x10^{-2} \pm 4.20x10^{-3}$ ^{c}	$3.31x10^{-2} \pm 3.73x10^{-3}$ ^{a}	$1.86x10^{-2} \pm 6.21x10^{-3}$ ^{b}	$1.75x10^{-2} \pm 8.26x10^{-3}$ ^{bc}
Proline	$3.57x10^{-2} \pm 8.86x10^{-3}$ ^{b}	$6.46x10^{-2} \pm 2.17x10^{-2}$ ^{a}	$5.56x10^{-2} \pm 1.09x10^{-2}$ ^{a}	$2.12x10^{-2} \pm 8.44x10^{-3}$ ^{b}
Lactate	$2.37x10^{-2} \pm 7.34x10^{-3}$ ^{a}	$1.25x10^{-2} \pm 4.89x10^{-3}$ ^{b}	$1.83x10^{-2} \pm 6.55x10^{-3}$ ^{ab}	$2.95x10^{-2} \pm 1.45x10^{-2}$ ^{a}
Trehalose	$2.89x10^{-2} \pm 2.91x10^{-2}$ ^{b}	$8.64x10^{-1} \pm 3.91x10^{-1}$ ^{a}	$5.40x10^{-1} \pm 3.90x10^{-1}$ ^{a}	$1.53x10^{-2} \pm 4.39x10^{-3}$ ^{b}
Glycerol	$1.14x10^{+0} \pm 5.04x10^{-1}$ ^{a}	$6.81x10^{-1} \pm 5.63x10^{-1}$ ^{b}	$6.31x10^{-1} \pm 4.28x10^{-1}$ ^{ab}	$3.68x10^{-1} \pm 7.93x10^{-2}$ ^{b}
Glucose	$5.08x10^{-2} \pm 7.57x10^{-2}$ ^{b}	$2.05x10^{-1} \pm 1.18x10^{-1}$ ^{a}	$5.55x10^{-1} \pm 6.75x10^{-1}$ ^{a}	$1.08x10^{-2} \pm 4.22x10^{-3}$ ^{b}

O-Phosphocholine	$3.91x10^{-3} \pm 3.42x10^{-3}$ ^{bc}	$7.92x10^{-3} \pm 2.05x10^{-3}$ ^{a}	$5.99x10^{-3} \pm 1.64x10^{-3}$ ^{ab}	$2.41x10^{-3} \pm 1.45x10^{-3}$ ^{c}
Ornithine	$3.35x10^{-3} \pm 2.46x10^{-3}$ ^{b}	$8.24x10^{-3} \pm 6.89x10^{-3}$ ^{ab}	$1.23x10^{-2} \pm 6.90x10^{-3}$ ^{a}	$9.03x10^{-3} \pm 5.34x10^{-3}$ ^{ab}
Lysine	$3.50x10^{-2} \pm 1.21x10^{-2}$ ^{c}	$4.48x10^{-2} \pm 9.29x10^{-3}$ ^{bc}	$9.28x10^{-2} \pm 6.41x10^{-2}$ ^{a}	$8.17x10^{-2} \pm 1.22x10^{-2}$ ^{ab}
Asparagine	$2.26x10^{-2} \pm 6.19x10^{-3}$ ^{b}	$3.24x10^{-2} \pm 6.98x10^{-3}$ ^{a}	$2.18x10^{-2} \pm 6.43x10^{-3}$ ^{b}	$2.49x10^{-2} \pm 2.63x10^{-3}$ ^{ab}
Aspartate	$3.33x10^{-2} \pm 1.37x10^{-2}$ ^{c}	$5.78x10^{-2} \pm 1.20x10^{-2}$ ^{b}	$3.81x10^{-2} \pm 1.23x10^{-2}$ ^{bc}	$9.78x10^{-2} \pm 2.00x10^{-2}$ ^{a}
Malate	$4.01x10^{-2} \pm 9.92x10^{-3}$ ^{b}	$5.43x10^{-2} \pm 1.04x10^{-2}$ ^{a}	$5.74x10^{-2} \pm 5.15x10^{-3}$ ^{a}	$7.47x10^{-2} \pm 1.10x10^{-2}$ ^{a}
Methionine	$9.61x10^{-3} \pm 3.80x10^{-3}$ ^{b}	$1.44x10^{-2} \pm 4.21x10^{-3}$ ^{a}	$1.40x10^{-2} \pm 4.94x10^{-3}$ ^{ab}	$8.15x10^{-3} \pm 1.74x10^{-3}$ ^{b}
Citrate	$3.34x10^{-2} \pm 4.83x10^{-3}$ ^{b}	$3.49x10^{-2} \pm 5.66x10^{-3}$ ^{ab}	$4.13x10^{-2} \pm 7.55x10^{-3}$ ^{a}	$3.75x10^{-2} \pm 1.21x10^{-3}$ ^{ab}
Glutamine	$3.73x10^{-2} \pm 1.82x10^{-2}$ ^{b}	$1.22x10^{-1} \pm 4.35x10^{-2}$ ^{a}	$5.86x10^{-2} \pm 3.95x10^{-2}$ ^{b}	$5.11x10^{-2} \pm 1.12x10^{-2}$ ^{b}
2-Oxoglutarate	$4.01x10^{-3} \pm 8.68x10^{-4}$ ^{b}	$7.64x10^{-3} \pm 3.90x10^{-3}$ ^{a}	$3.94x10^{-3} \pm 8.90x10^{-4}$ ^{b}	$3.32x10^{-3} \pm 5.81x10^{-4}$ ^{b}
Pyroglutamate	$8.13x10^{-3} \pm 1.40x10^{-3}$ ^{a}	$7.72x10^{-3} \pm 2.31x10^{-3}$ ^{a}	$7.24x10^{-3} \pm 3.80x10^{-3}$ ^{ab}	$4.78x10^{-3} \pm 1.76x10^{-3}$ ^{b}
Succinate	$1.86x10^{-2} \pm 5.03x10^{-3}$ ^{a}	$1.33x10^{-2} \pm 2.12x10^{-3}$ ^{b}	$8.99x10^{-3} \pm 2.59x10^{-3}$ ^{c}	$1.72x10^{-2} \pm 5.41x10^{-3}$ ^{ab}
Pyruvate	$2.52x10^{-2} \pm 1.05x10^{-2}$ ^{a}	$3.06x10^{-2} \pm 1.16x10^{-2}$ ^{a}	$7.97x10^{-3} \pm 2.20x10^{-3}$ ^{b}	$6.92x10^{-3} \pm 4.06x10^{-3}$ ^{b}
Glutamate	$6.61x10^{-2} \pm 3.53x10^{-2}$ ^{b}	$2.60x10^{-1} \pm 5.45x10^{-2}$ ^{a}	$1.76x10^{-1} \pm 5.36x10^{-2}$ ^{a}	$1.90x10^{-1} \pm 5.29x10^{-2}$ ^{a}
Acetone	$1.67x10^{-2} \pm 8.48x10^{-3}$ ^{a}	$5.07x10^{-3} \pm 4.43x10^{-3}$ ^{b}	$5.19x10^{-3} \pm 4.08x10^{-3}$ ^{b}	$1.74x10^{-2} \pm 1.06x10^{-2}$ ^{a}
Acetate	$1.04x10^{+0} \pm 4.77x10^{-1}$ ^{a}	$4.03x10^{-1} \pm 1.78x10^{-1}$ ^{b}	$2.53x10^{-1} \pm 1.30x10^{-1}$ ^{b}	$2.72x10^{-1} \pm 1.42x10^{-1}$ ^{b}
3-Methyl-2-oxovalerate	$5.47x10^{-2} \pm 1.24x10^{-2}$ ^{b}	$7.57x10^{-2} \pm 7.22x10^{-3}$ ^{a}	$7.82x10^{-2} \pm 1.92x10^{-2}$ ^{a}	$7.11x10^{-2} \pm 1.05x10^{-2}$ ^{ab}
Alanine	$1.30x10^{-1} \pm 5.37x10^{-2}$ ^{b}	$1.78x10^{-1} \pm 5.95x10^{-2}$ ^{ab}	$2.67x10^{-1} \pm 1.06x10^{-1}$ ^{a}	$1.34x10^{-1} \pm 1.68x10^{-2}$ ^{ab}
Acetoin	$1.20x10^{-2} \pm 3.52x10^{-3}$ ^{a}	$4.39x10^{-3} \pm 1.50x10^{-3}$ ^{b}	$8.74x10^{-3} \pm 4.87x10^{-3}$ ^{a}	$9.61x10^{-3} \pm 1.81x10^{-3}$ ^{a}
Ethanol	$3.29x10^{+0} \pm 1.16x10^{+0}$ ^{a}	$1.80x10^{+0} \pm 6.43x10^{-1}$ ^{b}	$1.53x10^{+0} \pm 4.66x10^{-1}$ ^{b}	$3.06x10^{+0} \pm 1.75x10^{+0}$ ^{ab}
2,3-Butanediol	$9.67x10^{-3} \pm 2.42x10^{-3}$ ^{a}	$3.52x10^{-3} \pm 1.13x10^{-3}$ ^{b}	$4.83x10^{-3} \pm 2.73x10^{-3}$ ^{b}	$1.13x10^{-2} \pm 4.00x10^{-3}$ ^{a}
Propionate	$1.33x10^{-1} \pm 2.72x10^{-2}$ ^{a}	$6.50x10^{-2} \pm 3.23x10^{-2}$ ^{b}	$6.40x10^{-2} \pm 2.91x10^{-2}$ ^{b}	$7.52x10^{-2} \pm 5.28x10^{-3}$ ^{b}
Isoleucine	$1.18x10^{-2} \pm 2.52x10^{-3}$ ^{b}	$1.59x10^{-2} \pm 2.28x10^{-3}$ ^{a}	$1.44x10^{-2} \pm 5.11x10^{-3}$ ^{ab}	$1.76x10^{-2} \pm 3.20x10^{-3}$ ^{a}
Valine	$2.17x10^{-2} \pm 4.01x10^{-3}$ ^{b}	$3.87x10^{-2} \pm 7.38x10^{-3}$ ^{a}	$3.47x10^{-2} \pm 6.72x10^{-3}$ ^{a}	$3.82x10^{-2} \pm 7.81x10^{-3}$ ^{a}
2-Aminobutyrate	$4.77x10^{-3} \pm 1.02x10^{-3}$ ^{b}	$4.21x10^{-3} \pm 6.90x10^{-4}$ ^{b}	$7.23x10^{-3} \pm 2.05x10^{-3}$ ^{a}	$4.70x10^{-3} \pm 6.60x10^{-4}$ ^{b}
Leucine	$6.52x10^{-2} \pm 1.78x10^{-2}$ ^{b}	$9.43x10^{-2} \pm 1.52x10^{-2}$ ^{a}	$8.98x10^{-2} \pm 3.67x10^{-2}$ ^{ab}	$7.40x10^{-2} \pm 1.40x10^{-2}$ ^{ab}

[*] Compact letters display of the comparisons between each pair of groups, where two mean values followed by a common superscript are not significantly different.

Table S6: Concentration (mmol/L, mean ± sd) of extracellular metabolites significantly different between *C. albicans* sub-groups expressed as difference from growth medium.

	subgroup-1	subgroup-2	p-value
Tryptophan	$-2.91x10^{-3} \pm 1.05x10^{-2}$	$-3.86x10^{-2} \pm 3.84x10^{-2}$	$1.14x10^{-3}$
Cytidine	$-2.40x10^{-3} \pm 1.09x10^{-3}$	$-7.92x10^{-4} \pm 1.55x10^{-3}$	$4.96x10^{-2}$
Guanosine	$-2.30x10^{-3} \pm 9.15x10^{-5}$	$-1.57x10^{-3} \pm 1.22x10^{-3}$	$6.92x10^{-3}$
Hydroxyacetone	$2.53x10^{-3} \pm 6.79x10^{-4}$	$4.54x10^{-3} \pm 2.23x10^{-3}$	$2.36x10^{-3}$
Arabinitol	1.02 ± 0.68	2.02 ± 0.11	$5.00x10^{-2}$
Sarcosine	$4.67x10^{-3} \pm 1.40x10^{-3}$	$5.65x10^{-4} \pm 3.71x10^{-3}$	$1.81x10^{-3}$
Malate	$-3.64x10^{-2} \pm 7.50x10^{-3}$	$-1.32x10^{-2} \pm 2.63x10^{-2}$	$2.10x10^{-3}$
2-Oxoglutarate	$5.58x10^{-2} \pm 3.15x10^{-2}$	$1.17x10^{-1} \pm 7.27x10^{-2}$	$1.81x10^{-2}$
Acetone	$-4.58x10^{-3} \pm 9.10x10^{-4}$	$-3.04x10^{-3} \pm 2.55x10^{-3}$	$4.26x10^{-2}$
Methionine	$-3.78x10^{-2} \pm 6.24x10^{-3}$	$-6.76x10^{-2} \pm 3.94x10^{-2}$	$1.58x10^{-3}$
Acetate	$1.72x10^{-1} \pm 3.14x10^{-1}$	$7.67x10^{-1} \pm 5.60x10^{-1}$	$1.91x10^{-2}$
Alanine	$9.55x10^{-2} \pm 2.50x10^{-2}$	$-2.68x10^{-4} \pm 1.03x10^{-1}$	$6.45x10^{-4}$
Acetoin	$9.37x10^{-2} \pm 3.54x10^{-2}$	$1.80x10^{-1} \pm 8.73x10^{-2}$	$5.61x10^{-3}$
Leucine	$9.33x10^{-2} \pm 3.18x10^{-2}$	$-2.83x10^{-2} \pm 1.08x10^{-1}$	$2.92x10^{-4}$

Table S7: Concentration (mmol/L, mean ± sd) of intracellular metabolites significantly different between *C. albicans* sub-groups

	subgroup-1	subgroup-2	p-value
Fumarate	$3.12x10^{-3} \pm 1.64x10^{-4}$	$3.67x10^{-3} \pm 4.79x10^{-4}$	$7.49x10^{-4}$
Trehalose	$1.37x10^{-2} \pm 2.47x10^{-3}$	$2.96x10^{-2} \pm 3.00x10^{-2}$	$1.55x10^{-2}$
Glycerol	1.66 ± 0.18	1.06 ± 0.50	$8.21x10^{-4}$
Glucose	$6.30x10^{-3} \pm 5.88x10^{-3}$	$5.97x10^{-2} \pm 8.02x10^{-2}$	$3.06x10^{-3}$
O-Phosphocholine	$1.98x10^{-3} \pm 3.64x10^{-4}$	$4.12x10^{-3} \pm 3.63x10^{-3}$	$8.29x10^{-3}$
Glutamine	$2.05x10^{-2} \pm 1.59x10^{-3}$	$3.94x10^{-2} \pm 1.84x10^{-2}$	$3.39x10^{-5}$
Pyroglutamate	$6.82x10^{-3} \pm 8.06x10^{-4}$	$8.39x10^{-3} \pm 1.37x10^{-3}$	$1.68x10^{-2}$
4-Aminobutyrate	$5.15x10^{-3} \pm 1.53x10^{-4}$	$6.66x10^{-3} \pm 1.83x10^{-3}$	$4.35x10^{-4}$
Ethanol	4.59 ± 0.28	3.05 ± 1.12	$1.06x10^{-5}$
2-Aminobutyrate	$4.06x10^{-3} \pm 4.48x10^{-4}$	$4.89x10^{-3} \pm 1.06x10^{-3}$	$2.35\ x10^{-2}$

Table S8. Mean concentration in mmol/L (sd) of extracellular metabolites significantly different between *Candida* biofilm categories.

	0	1	2	3	p-value
2-Oxoisocaproate	2.03x10-02 (1.83x10-02)	3.53x10-02 (2.27x10-02)	2.43x10-02 (6.60x10-02)	6.82x10-02 (1.29x10-02)	1.08x10-04
Orotate	1.59x10-03 (8.43x10-04)	1.59x10-03 (5.25x10-04)	1.18x10-03 (9.32x10-04)	5.43x10-04 (1.88x10-04)	1.86x10-03
Inosine	2.14x10-03 (5.44x10-03)	1.13x10-03 (2.43x10-03)	-3.53x10-04 (1.29x10-03)	-1.15x10-03 (6.98x10-04)	7.99x10-03
Choline	-2.00x10-02 (1.15x10-02)	-2.02x10-02 (1.71x10-02)	-2.50x10-02 (1.32x10-02)	-2.58x10-02 (1.35x10-03)	1.20x10-02
Lysine	1.22x10-01 (5.35x10-02)	1.23x10-01 (5.67x10-02)	7.11x10-02 (6.31x10-02)	8.07x10-02 (4.74x10-02)	4.56x10-02

0: no biofilm; 1: weak producers; 2 intermediate producers; 3 strong producers

Table S9. Mean concentration in mmol/L (sd) of intracellular metabolites significantly different between *Candida* biofilm categories.

	0	1	2	3	p-value
Ornithine	2.97x10-03 (2.35x10-03)	4.00x10-03 (4.84x10-03)	4.37x10-03 (1.83x10-03)	1.00x10-02 (1.82x10-03)	2.49x10-04
Malate	3.68x10-02 (2.15x10-02)	4.89x10-02 (2.05x10-02)	4.59x10-02 (2.18x10-02)	6.06x10-02 (1.27x10-02)	4.80x10-04
2-Oxoglutarate	4.62x10-03 (1.21x10-03)	4.25x10-03 (9.50x10-04)	4.25x10-03 (1.39x10-03)	3.45x10-03 (3.28x10-04)	2.61x10-03
Pyruvate	3.11x10-02 (1.20x10-02)	2.41x10-02 (1.23x10-02)	1.65x10-02 (1.43x10-02)	9.46x10-03 (5.61x10-03)	7.30x10-03
UMP	8.04x10-03 (2.50x10-03)	1.07x10-02 (3.03x10-03)	1.13x10-02 (1.30x10-03)	1.52x10-02 (2.05x10-03)	1.67x10-02
Glutamate	4.72x10-02 (1.88x10-01)	9.63x10-02 (1.49x10-01)	9.72x10-02 (1.06x10-01)	1.50x10-01 (3.15x10-02)	3.07x10-02
AMP	1.16x10-02 (6.68x10-03)	1.41x10-02 (9.47x10-03)	1.41x10-02 (3.90x10-03)	1.93x10-02 (5.38x10-03)	4.20x10-02
Lysine	2.97x10-02 (2.33x10-02)	4.12x10-02 (1.94x10-02)	4.56x10-02 (2.14x10-02)	5.96x10-02 (2.65x10-02)	4.56x10-02
Glycerol	1.28(1.19)	1.23 (8.47x10-01)	7.48x10-01 (6.05x10-01)	4.24x10-01 (1.69x10-01)	4.88x10-02

0: no biofilm; 1: weak producers; 2 intermediate producers; 3 strong producers

Table S10. Mean concentration in mmol/L (sd) of extracellular metabolites significantly different between fluconazole-resistant (R) and susceptible (S) *Candida* strains.

	R	S	p-value
Isoleucine	-7.33x10-02 (2.29x10-02)	-3.67x10-02 (1.67x10-02)	5.18x10-06
Sarcosine	-7.54x10-03 (8.74x10-03)	1.67x10-03 (4.31x10-03)	2.60x10-05
Inosine	3.31x10-03 (5.23x10-03)	1.54x10-05 (1.96x10-03)	2.58x10-04
Asparagine	-1.47x10-01 (6.30x10-02)	-1.73x10-01 (1.35x10-02)	1.76x10-03
Acetate	-1.27x10-01 (4.68x10-01)	5.09x10-01 (9.90x10-01)	2.22x10-03
Fumarate	1.29x10-02 (8.68x10-03)	2.64x10-02 (2.12x10-02)	2.79x10-03
Arabinitol	-6.87x10-03 (1.95)	1.63 (1.35)	2.93x10-03
Leucine	-1.35x10-01 (1.26x10-01)	8.69x10-03 (1.66x10-01)	3.01x10-03
Alloisoleucine	6.31x10-01 (2.77x10-01)	3.73x10-01 (1.80x10-01)	3.91x10-03
Choline	-8.94x10-03 (5.37x10-02)	-2.37x10-02 (9.53x10-03)	3.96x10-03
Guanosine	1.90x10-03 (5.19x10-03)	-2.05x10-03 (2.53x10-03)	5.30x10-03
Methionine	-1.19x10-01 (5.57x10-02)	-5.65x10-02 (5.82x10-02)	5.75x10-03
Phenylalanine	1.27x10-02 (4.30x10-02)	6.18x10-02 (4.42x10-02)	7.53x10-03
Cytidine	2.21x10-03 (6.43x10-03)	-8.24x10-04 (2.92x10-03)	8.18x10-03
Succinate	5.29x10-01 (2.10x10-01)	3.40x10-01 (2.65x10-01)	1.01x10-02
Acetoin	7.77x10-02 (1.10x10-01)	1.86x10-01 (1.44x10-01)	1.02x10-02
Formate	-1.81x10-02 (2.68x10-02)	1.22x10-02 (4.77x10-02)	1.06x10-02
Malate	1.14x10-02 (6.26x10-02)	-2.76x10-02 (3.22x10-02)	1.70x10-02
3-Methyl-2-oxovalerate	4.70x10-02 (1.60x10-02)	3.15x10-02 (1.37x10-02)	2.76x10-02
N6-Acetyllysine	-8.36x10-02 (4.77x10-02)	-4.29x10-02 (3.43x10-02)	2.99x10-02
Glucose	-3.89x10+01 (1.10x10+01)	-3.15x10+01 (1.01x10+01)	3.86x10-02
Ethanol	1.02x10+02 (1.88x10+01)	8.32x10+01 (2.94x10+01)	4.87x10-02

Table S11. Mean concentration in mmol/L (sd) of intracellular metabolites significantly different between fluconazole-resistant (R) and susceptible (S) *Candida* strains.

	R	S	p-value
Glutamate	2.18x10-01 (1.22x10-01)	6.38x10-02 (6.77x10-02)	1.05x10-05
Glutamine	9.97x10-02 (7.09x10-02)	3.27x10-02 (1.94x10-02)	1.92x10-05
Threonine	2.77x10-02 (1.65x10-02)	1.33x10-02 (4.19x10-03)	5.60x10-05
Acetone	4.00x10-03 (4.28x10-03)	1.52x10-02 (1.28x10-02)	1.71x10-04
Acetoin	5.92x10-03 (3.41x10-03)	1.17x10-02 (5.37x10-03)	2.49x10-04
AMP	1.92x10-02 (6.30x10-03)	1.24x10-02 (5.93x10-03)	2.97x10-04
Asparagine	3.13x10-02 (1.25x10-02)	2.17x10-02 (6.55x10-03)	4.84x10-04
Aspartate	5.81x10-02 (1.37x10-02)	2.95x10-02 (1.80x10-02)	9.38x10-04
Adenosine	1.21x10-02 (4.58x10-03)	8.29x10-03 (3.13x10-03)	1.60x10-03
Tyrosine	1.27x10-02 (2.60x10-03)	9.83x10-03 (3.96x10-03)	1.69x10-03
Propionate	6.93x10-02 (7.67x10-02)	1.26x10-01 (5.11x10-02)	1.76x10-03
Trehalose	6.95x10-01 (8.09x10-01)	2.03x10-02 (5.63x10-02)	2.19x10-03
2,3-Butanediol	4.57x10-03 (4.02x10-03)	9.53x10-03 (2.57x10-03)	2.60x10-03
2-Oxoglutarate	4.96x10-03 (2.18x10-03)	3.95x10-03 (1.16x10-03)	2.95x10-03
O-Phosphocholine	6.90x10-03 (4.13x10-03)	2.61x10-03 (3.02x10-03)	4.51x10-03
Glycerol	4.07x10-01 (4.28x10-01)	1.23x10+00 (8.20x10-01)	4.82x10-03
Valine	3.42x10-02 (1.72x10-02)	2.32x10-02 (6.95x10-03)	5.36x10-03
Ethanol	2.07 (1.08)	3.17 (1.90)	8.11x10-03
Ornithine	4.96x10-03 (1.14x10-02)	3.68x10-03 (2.89x10-03)	3.29x10-02
Malate	5.37x10-02 (1.23x10-02)	4.25x10-02 (1.84x10-02)	3.38x10-02
Proline	4.56x10-02 (3.24x10-02)	3.75x10-02 (6.10x10-03)	3.39x10-02
Glucose-1-phosphate	1.48x10-02 (1.93x10-02)	2.84x10-02 (3.48x10-02)	4.21x10-02
Isoleucine	1.49x10-02 (5.90x10-03)	1.22x10-02 (3.80x10-03)	4.83x10-02
3-Methyl-2-oxovalerate	7.20x10-02 (1.46x10-02)	6.19x10-02 (2.34x10-02)	4.92x10-02

Table S12. Mean concentration in mmol/L (sd) of extracellular metabolites significantly different between caspofungin-resistant (R) and susceptible (S) *Candida* strains.

	R	S	p-value
Putrescine	5.35x10-03 (4.37x10-03)	-3.16x10-03 (9.73x10-03)	3.06x10-05
Pyruvate	1.28 (1.04)	6.02 (4.36)	1.25x10-04
Glucose	-24.4 (10.5)	-35.6 (15.9)	1.48x10-04
Ribose	-4.47x10-02 (2.25x10-02)	-7.22x10-02 (3.60x10-02)	3.24x10-04
Methionine	-2.65x10-02 (2.52x10-02)	-8.71x10-02 (7.88x10-02)	3.32x10-04
Choline	-3.00x10-02 (1.50x10-03)	-1.94x10-02 (1.16x10-02)	3.68x10-04
Mannose	6.57x10-02 (3.66x10-02)	1.33x10-02 (2.28x10-02)	3.89x10-03
2-Oxoisocaproate	1.13x10-01 (5.56x10-02)	2.47x10-02 (3.09x10-02)	5.69x10-03
Arabinitol	3.00 (1.80)	1.25 (1.83)	7.57x10-03
Methanol	-9.72x10-03 (1.87x10-03)	-4.22x10-03 (4.64x10-03)	8.05x10-03
2,3-Butanediol	5.97x10-02 (6.61x10-02)	2.27x10-01 (1.14x10-01)	9.36x10-03
Alanine	5.01x10-01 (3.34x10-01)	6.74x10-02 (1.42x10-01)	1.06x10-02
p-cresol	6.61x10-03 (2.47x10-03)	3.27x10-03 (3.58x10-03)	1.07x10-02
Succinate	9.85x10-02 (3.94x10-02)	4.54x10-01 (2.34x10-01)	1.40x10-02
Ethanol	53.3 (31.8)	9.24x10+01 (26.7)	2.18x10-02
Sarcosine	7.20x10-04 (2.37x10-03)	-9.87x10-04 (8.82x10-03)	2.65x10-02
Hydroxyacetone	1.14x10-03 (1.13x10-03)	4.46x10-03 (3.91x10-03)	3.66x10-02
Phenylalanine	-3.35x10-02 (5.90x10-02)	5.37x10-02 (4.73x10-02)	4.21x10-02
Glycerol	1.58 (4.90x10-01)	2.43x10+00 (1.19)	4.46x10-02

Table S13. Mean concentration in mmol/L (sd) of intracellular metabolites significantly different between caspofungin-resistant (R) and susceptible (S) *Candida* strains.

	R	S	p-value
Pyruvate	9.53x10-03 (2.65x10-03)	2.44x10-02 (1.62x10-02)	8.68x10-06
Glutamate	1.74x10-01 (5.95x10-02)	7.36x10-02 (1.36x10-01)	3.58x10-05
Malate	5.71x10-02 (6.18x10-03)	4.53x10-02 (1.85x10-02)	1.25x10-04
Acetate	2.88x10-01 (1.79x10-01)	8.32x10-01 (7.85x10-01)	5.75x10-04
UMP	1.46x10-02 (2.35x10-03)	1.05x10-02 (3.87x10-03)	6.29x10-04
Ornithine	1.09x10-02 (5.37x10-03)	3.48x10-03 (2.57x10-03)	1.08x10-03
Alanine	2.33x10-01 (6.20x10-02)	1.28x10-01 (7.09x10-02)	2.83x10-03
Sucrose	6.44x10-03 (3.21x10-03)	1.18x10-02 (7.59x10-03)	3.18x10-03
AMP	1.90x10-02 (4.65x10-03)	1.32x10-02 (7.15x10-03)	4.32x10-03
Trehalose	3.59x10-01 (4.60x10-01)	2.07x10-02 (9.43x10-02)	9.77x10-03
O-Phosphocholine	5.96x10-03 (2.30x10-03)	3.05x10-03 (4.74x10-03)	1.12x10-02
3-Methyl-2-oxovalerate	6.91x10-02 (1.20x10-02)	6.35x10-02 (2.52x10-02)	3.05x10-02
Acetone	4.02x10-03 (4.55x10-03)	1.40x10-02 (1.58x10-02)	3.42x10-02
Uracil	5.84x10-03 (1.55x10-03)	7.12x10-03 (1.92x10-03)	4.31x10-02
Phenylalanine	1.76x10-02 (2.38x10-03)	2.09x10-02 (5.70x10-03)	4.48x10-02
Succinate	8.13x10-03 (4.60x10-03)	1.67x10-02 (7.65x10-03)	4.76x10-02

Table S14. Mean concentration in mmol/L (sd) of extracellular metabolites significantly different between SAP producers (P) and non-producers (N).

	N	P	p-value
Arabinitol	9.18x10-01 (1.63x10+00)	1.94x10+00 (1.26x10+00)	1.69x10-04
Valine	-1.12x10-02 (4.78x10-02)	-3.11x10-02 (2.81x10-02)	8.75x10-03
Inosine	1.14x10-03 (4.15x10-03)	-4.61x10-05 (2.47x10-03)	1.10x10-02
Malate	-3.67x10-03 (5.13x10-02)	-2.90x10-02 (3.02x10-02)	1.25x10-02
Choline	-1.80x10-02 (1.96x10-02)	-2.45x10-02 (8.68x10-03)	1.60x10-02
Sucrose	1.89x10-02 (8.65x10-03)	1.34x10-02 (3.93x10-03)	1.63x10-02
Uracil	4.29x10-03 (6.39x10-03)	5.15x10-03 (3.08x10-03)	2.23x10-02
Lactate	2.24x10-02 (6.68x10-02)	4.14x10-02 (8.14x10-02)	4.12x10-02
Acetoin	9.76x10-02 (1.55x10-01)	2.09x10-01 (7.73x10-02)	4.20x10-02
N6-Acetyllysine	-6.07x10-02 (4.15x10-02)	-4.29x10-02 (4.04x10-02)	5.00x10-02

Table S15. Mean concentration in mmol/L (sd) of intracellular metabolites significantly different between SAP producers (P) and non-producers (N).

	N	P	p-value
Threonine	1.48 x10-02 (1.62x10-02)	1.32x10-02 (7.85x10-03)	2.02x10-02
Valine	2.65x10-02 (1.32x10-02)	2.20x10-02 (1.16x10-02)	4.63x10-02
Pyruvate	2.45x10-02 (1.58x10-02)	1.60x10-02 (1.41x10-02)	4.79x10-02
